# Properties of a Random Bipartite Geometric Associator Graph Inspired by Vehicular Networks

**DOI:** 10.3390/e25121619

**Published:** 2023-12-04

**Authors:** Kaushlendra Pandey, Abhishek K. Gupta, Harpreet S. Dhillon, Kanaka Raju Perumalla

**Affiliations:** 1 Department of Electrical Engineering, Indian Institute of Technology Kanpur, Kanpur 208016, India; kpandey@iitk.ac.in (K.P.); gkrabhi@iitk.ac.in (A.K.G.); pkraju@iitk.ac.in (K.R.P.); 2 Wireless@VT, Bradley Department of Electrical and Computer Engineering, Virginia Tech, Blacksburg, VA 24061, USA

**Keywords:** Poisson line process, Poisson point process, Cox process, load distribution in vehicular communication, vehicular network

## Abstract

We consider a point process (PP) generated by superimposing an independent Poisson point process (PPP) on each line of a 2D Poisson line process (PLP). Termed PLP-PPP, this PP is suitable for modeling networks formed on an irregular collection of lines, such as vehicles on a network of roads and sensors deployed along trails in a forest. Inspired by vehicular networks in which vehicles connect with their nearest wireless base stations (BSs), we consider a *random bipartite associator graph* in which each point of the PLP-PPP is associated with the nearest point of an independent PPP through an edge. This graph is equivalent to the partitioning of PLP-PPP by a Poisson Voronoi tessellation (PVT) formed by an *independent* PPP. We first characterize the exact distribution of the number of points of PLP-PPP falling inside the ball centered at an arbitrary location in $R^{2}$ as well as the typical point of PLP-PPP. Using these distributions, we derive cumulative distribution functions (CDFs) and probability density functions (PDFs) of *k*th contact distance (CD) and the nearest neighbor distance (NND) of PLP-PPP. As intermediate results, we present the empirical distribution of the perimeter and approximate distribution of the length of the typical chord of the zero-cell of this PVT. Using these results, we present two close approximations of the distribution of node degree of the random bipartite associator graph. In a vehicular network setting, this result characterizes the number of vehicles connected to each BS, which models its *load*. Since each BS has to distribute its limited resources across all the vehicles connected to it, a good statistical understanding of load is important for an efficient system design. Several applications of these new results to different wireless network settings are also discussed.

## 1. Introduction

### 1.1. Background and Motivation

To provide control and connectivity to a network of devices, a set of control/access nodes are often deployed that form a control/communication network where each device (acting as a user node) is connected to one control/access node (acting as a master node). Consider, for instance, a wireless cellular network consisting of several base stations (BSs) providing connectivity to a set of mobile users, where each user is connected to a BS to receive channel access control and exchange data. Which user connects with which BS (termed cell or BS association) is a function of the network geometry (since users usually connect with their proximate BSs). A specific example of this setting is when each user node is associated with its closest master node. The association between these two types of nodes can be represented using a simple bipartite many-to-one associator graph with edges from the user nodes to master nodes with each edge representing an association.

Given the natural irregularity in the node locations, tools from stochastic geometry (SG) have been used extensively to model and analyze such networks. The underlying idea is to model the locations of these two types of nodes as realizations of appropriately selected point processes (PPs) [1]. Therefore, the associator graph on this network can be thought of as a variant of *AB random geometric graph* and is hence termed the *random bipartite geometric associator graph* in this paper. As we will discuss in the sequel, the properties of this graph are of key importance in the modeling and analysis of control/communication networks. For instance, the degree of a master node provides the number of user nodes associated with this master node, which we term as its *load*. Since the amount of resources allocated by a master node to each of its user nodes will depend upon its load (such as bandwidth assigned to each user in the cellular network example above), it is easy to deduce that the load directly impacts the performance of each user node.

Before going further, it is useful to note that models from stochastic geometry (such as point and line processes) have also been used extensively in statistical physics. Therefore, one can find numerous examples of PPs and related models that were originally inspired by networks but later found interest in the statistical physics community, such as [2,3,4,5,6,7,8]. Our hope is that the current contribution will also lie in the same category. In general, SG has found applications in many diverse fields, such as forestry, geophysics, economics, biology, and telecommunications, e.g., see [1,9,10,11,12] for a small sample.

### 1.2. PLP-PPP Random Bipartite Geometric Associator Graph

Now we introduce the main object of this paper, which is inspired by vehicular networks. We first describe the underlying PP of interest using which the random associator graph will be constructed.

If we consider a single road, the locations of vehicles on a road can be modeled using a Poisson point process (PPP). However, a general vehicular network consists of *multiple* vehicles distributed on *multiple* roads located throughout the city. A popular model for this setting involves modeling the underlying road network as a Poisson line process (PLP) and then distributing vehicles on each road as a 1D PPP. Since conditioned on the road locations, the vehicles form a PPP with density determined by the realization of the roads (equivalently the PLP), the overall process is a Cox process with density driven by a PLP [10]. We term this doubly-stochastic point process as a PLP-driven-Poisson Cox point process or a PLP-PPP in short [10]. It was first presented in [13] to model a vehicular mobile communication network and was studied comprehensively in [10].

We now enrich this model by considering an overlaid cellular network of BSs (modeled as an independent 2D PPP), such that each vehicle from the PLP-PPP is associated with the closest BS from this PPP (which will also maximize its received signal-to-noise-plus-interference ratio (SINR)). Such wireless links between vehicles and the BSs are termed vehicle-to-infrastructure (V2I) links. This type of geometry based association results in an interesting random bipartite geometric associator graph where the nodes from PLP-PPP (representing vehicles) connect to the nearest control nodes forming an independent PPP (representing BSs). We term this the *PLP-PPP Random Bipartite Geometric Associator Graph*, which is the main topic of this paper.

For completeness, note that the association can also be seen as a partition of PLP-PPP by an independent Poisson Voronoi (PV) tessellation. Here, the load on a master node is simply the number of user nodes lying in its PV cell. By definition, this is the same the node degree of a control/access node of the above graph. As discussed above already, the load on each BS will impact the amount of resources allocated to each vehicle and will hence impact the performance of this network. Using the PLP-PPP model, recent works have analyzed coverage probability [14,15,16,17,18], load distribution [19], and other such metrics for vehicular networks.

PLP-PPP can also be used to model other deployments along a set of random lines, e.g., sensors along the trails in a forest. Here, sensors can associate with a fusion node that collects their data. Assuming fusion nodes are distributed as a 2D PPP, the load on each fusion node is given by the node degree of the above graph. We will also consider a case study inspired by this setting.

Note that the PLP-PPP can be further generalized (or restricted) to model various variations of vehicular traffic. In [20], the authors captured platooned vehicular traffic by modeling vehicles on each line of a PLP by a Matérn cluster process, thereby giving rise to a PLP-MCP. As another example, if we restrict the orientation of roads to only two orthogonal directions, the line process reduces to a Manhattan line process (MLP), thereby giving rise to an MLP-PPP model of vehicular traffic. Although our focus in the paper is restricted to PLP-PPP, our results and derivations can also be applied to such variations of the PLP-PPP.

### 1.3. Context and Contributions

The focus of this paper is on studying several key properties of PLP-PPP and the above-described random bipartite geometric associator graph. We are specifically interested in the Laplace functional (LF), probability generating functional (PGFL), contact distance (CD), and nearest neighbor distance (NND) of this PP. In order to put this contribution in context, we briefly describe directly relevant prior work in this direction. The characteristic properties of PLP-PPP have been studied in [14,15,18,21]. In particular, in [21], authors presented the expressions for the density, CDFs of CD and NND, and LF of PLP-PPP along with their Palm counterparts. Note that the Palm distribution of a PP refers to its distribution conditioned on the occurrence of one of its points at a specific location. The distributions for the number of lines intersecting a convex body and some distances in PLP-PPP are presented in [14]. The asymptotic properties of PLP-PPP along with the void probability (the probability that no point is located in a given set) are presented in [15]. In [18], authors presented the CDF and PDF of CD and NND of PLP-PPP.

Despite these existing works, there are some knowledge gaps that this paper will attempt to fill. First, the *k*-th CD and NND distributions of PLP-PPP have not been reported in the literature for general values of k>1. Second, the load distribution analyses described in the literature have their own constraints and limitations in the sense that they cannot be easily extended to a general Cox process in which general 1D PPs are used to model vehicle locations on each line of a PLP. Further, the study of rate coverage reported in the literature is inadequate, and metrics such as meta distribution of rate coverage, which quantifies the rate coverage of an individual link for a specific realization, have not been presented for the PLP-PPP model. Inspired by these gaps, this paper provides a comprehensive treatment of PLP-PPP as well as the corresponding associator graph described in Section 1.2. We also investigate some applications of these new results to wireless communications networks. The specific contributions of this paper are summarized next.

1. We provide simplified PGFs of the number of points of the PLP-PPP falling in a ball centered at an arbitrary location from $R^{2}$ and at a randomly selected point of PLP-PPP. Using these results, we provide closed form expressions for the CDFs of *k*-th CD and NND of PLP-PPP.
2. We then derive the node-degree distribution of the typical and tagged control/access node of a random bipartite geometric associator graph that associates a PLP-PPP to an independent PPP (described in Section 1.2). We also provide approximate distributions for the same.
3. As a key intermediate result, we present the empirical PDF of the perimeter of the zero-cell of the Poisson Voronoi (PV) cell for this setup as well as an approximate distribution of the length of any randomly selected chord of the zero-cell.
4. Finally, we discuss several applications of the new node degree distribution result in wireless networks. Examples include the simple closed form expressions for load distribution, rate coverage, meta distribution of rate, and coverage as well as content caching analysis in vehicular networks. We also provided a direct application of the derived node degree result to the analysis of a wireless sensor network.

### 1.4. Notation

Now, we present the important notations that we use throughout the paper. A vector in $R^{2}$ and R is denoted by bold style letter (x) and bold *italic* style (x) with their norms ||x|| and |x|, respectively. A ball in 1D and 2D centered at x and x of radius *r* is denoted by $b_{1}\left(x,r\right)$ and $b_{2}\left(x,r\right)$, respectively. For a set A, |A| denotes its Lebesgue measure, for example $|b_{1}\left(o,r\right)|\;=\;2r$. For a PP Ψ, the notation Ψ(B) denotes the number of points of Ψ falling inside the set B. The PGF, CDF, and PDF of a random variable (RV) *X* is denoted by $P_{X}\left(\;\cdot \;\right)$, $F_{X}\left(\;\cdot \;\right)$ and $f_{X}\left(\;\cdot \;\right)$, respectively. The expected value and variance of RV *X* is denoted by E[X] and Var[X], respectively. The notation $f^{\left(k\right)}\left(x\right)$ denotes the *k*-th derivative of function *f* with respect to *x*. For a RV *X* with PGF $P_{X}\left(s\right)$, the mean and the variance can be computed as

(1) $$\begin{array}{c} E\left[X\right]={\left[P_{X}^{\left(1\right)}\left(s\right)\right]}_{s=1},\;\mathrm{Var}\left[X\right]={\left[P_{X}^{\left(2\right)}\left(s\right)\right]}_{s=1}+E\left[X\right]-{\left(E\left[X\right]\right)}^{2}. \end{array}$$

The PDF of a generalized Gamma RV *X* with parameters a,b, and *c* is

(2) $$\begin{array}{c} f_{X}\left(a,b,c,x\right)=g\left(a,b,c,x\right)\overset{\Delta }{=}ab^{c/a}{\left(\Gamma \left(c/a\right)\right)}^{-1}x^{c-1}e^{-bx^{a}},x\geq 0, \end{array}$$

with its mean and variance being $\frac{b^{-1/a}\Gamma \left((c+1)/a\right)}{\Gamma (c/a)}$ and $b^{-2/a}\left(\frac{(c+2)/a}{\Gamma (c/a)}-{\left(\frac{(c+1)/a}{\Gamma (c/a)}\right)}^{2}\right)$, respectively. The Faà di Bruno’s formula [22] states that the *k*-th derivative $\frac{d^{k}}{ds^{k}}\exp\left(h\left(s\right)\right)$ of exp(h(s)) with respect to *s* is given as

(3) $$\begin{array}{cc} \; & \frac{d^{k}}{ds^{k}}\exp\left(f\left(s\right)\right)=\exp\left(f(s)\right)\sum_{N_{k}}\frac{k!}{n_{1}!\cdots n_{k}!}{\left(f^{\left(1\right)}\left(s\right)/1!\right)}^{n_{1}}\cdots {\left(f^{\left(k\right)}\left(s\right)/k!\right)}^{n_{k}}, \end{array}$$

where the sum is over set $N_{k}$ consisting of all k-tuples $\left\{n_{1}\cdots n_{k}\right\}$ with $n_{i}\geq 0$ and $n_{1}+2n_{2}+\ldots +kn_{k}=k$. The notation l(ρ,ϕ) denotes a line in $R^{2}$, where ρ is the length of the normal from the origin to the line, and ϕ is the angle that the normal subtends from the x-axis in the counter-clockwise direction. The point (ρcosϕ,ρsinϕ) is the point on the line that is nearest to the origin, which is termed the base of line l(ρ,ϕ).

The line l(ρ,ϕ) can also be represented as an element (ρ,ϕ) in the representation space C≡R×[0,π). Let $T_{l}\left(\;\cdot \;\right)$ denote the transformation of l(0,0) to the l(ρ,ϕ) given as

(4) $$\begin{array}{c} T_{l}\left(x\right)=\left(\rho \cos\phi +x\sin\phi ,\rho \sin\phi -x\cos\phi \right), \end{array}$$

**Figure 1:**
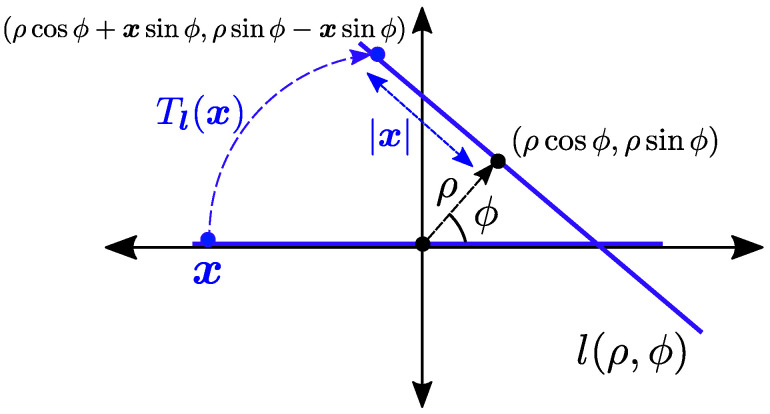
Illustration showing transformation of the point x located on x-axis to the line l(ρ,ϕ).

Here, $T_{l}\left(x\right)$ denotes the 2D location of a point located at the x-axis (i.e., l(0,0)) with coordinates (x,0) after getting transformed to a line l(ρ,ϕ) as seen in Figure 1. This also shows the absolute 2D location of a point of line l(ρ,ϕ) which is located at a distance *x* from the line’s base. The notation $P^{o}\left[\Phi \in P\right]$ denotes the Palm probability, which is the probability that the PP satisfies a property P conditioned on there being a point located at the origin. Further, $P^{o!}\left[\Phi \in P\right]$ denotes the reduced Palm probability which is the distribution of the PP excluding a point at the origin conditioned on the presence of that point at the origin, i.e., $P^{o!}\left[P\in \Phi \right]=P\left[\Phi \setminus \left\{o\right\}\in P|o\in \Phi \right]$.

## 2. System Model

In this work, the object of interest for us is a graph associating nodes of a PLP-PPP with the nodes of an independent PPP on a 2D space. In order to define this graph rigorously, we first provide the definitions of PLP and PLP-PPP.

**Definition** **1.** *(PLP) A random set $\Phi_{L}=\left\{l_{i}\left(\rho_{i},\phi_{i}\right),\;\forall i\in N\right\}$ of lines with $\rho_{i}$ and $\phi_{i}$ as the length and orientation of the perpendicular to the i-th line $l_{i}$ from the origin, is a PLP if the pairs $\left(\rho_{i},\phi_{i}\right)$ form a PPP in the representation space C≡R×[0,π).*

Note that an *i*-th line $l_{i}\left(\rho_{i},\phi_{i}\right)\in \Phi_{L}$ can be uniquely defined using the two parameters $\rho_{i}$ and $\phi_{i}$ which is essentially a 2D coordinate in the *representation space* C. Therefore, each line can be represented as a point in C, hence, a set of lines can be equivalently seen as a PP in C. The above definition says that a PLP is equivalently a PPP in C. Further, the PLP is characterized using the density parameter (denoted by $\lambda_{L}$) such that number of lines hitting a convex body K of perimeter P(K) is Poisson distributed with mean $\lambda_{L}P\left(K\right)$ [10].

**Definition** **2.** *(PLP-PPP) Let $\Phi_{L}=\left\{l_{i}\left(\rho_{i},\phi_{i}\right),\;\forall i\in N\right\}$ is a PLP with density $\lambda_{L}$. Let $\left\{\psi_{i},\;i\in N\right\}$ be independent and identically distributed PPPs in R with density $\lambda_{R}$. We assign i-th PPP $\psi_{i}$ to the i-th line $l_{i}\left(\rho_{i},\phi_{i}\right)$ and transform the points of $\psi_{i}$ to be on the line to get*

(5) $$\begin{array}{c} \Psi_{l_{i}}=\underset{x_{j,i}\in \psi_{i}}{⋃}\left\{x_{j,i}=T_{l_{i}}\left(x_{j,i}\right)\right\}, \end{array}$$

*where $T_{l}\left(\;\cdot \;\right)$ is defined in (4). Now, Consider a point process* Ψ *formed as the union of all the $\Psi_{l_{i}}$, i.e.,*

(6) $$\begin{array}{c} \Psi =\underset{l_{i}\in \Phi_{L}}{⋃}\Psi_{l_{i}}\;, \end{array}$$

*which includes all points located on each line of $\Phi_{L}$. The PP* Ψ *is a Cox process driven by PLP* [10]*. This has also been termed the Poisson line Cox process and also PLP-PPP.*

It is easy to check that Ψ is a stationary PP [21] with density $\mu =\pi \lambda_{L}\lambda_{R}$. Owing to stationarity, the analysis of the average properties of the PP can be performed by placing the typical point at the origin. In this work, we consider two types of nodes. The first set of nodes is distributed as a PLP-PPP Ψ with density μ in the $R^{2}$ space on a random network of lines, distributed as a PLP $\Phi_{L}$ with density $\lambda_{L}$. The second set of nodes is distributed as an independent 2D PPP $\Phi =\{y_{i}\}$ with density λ on the same space.

**Figure 2:**
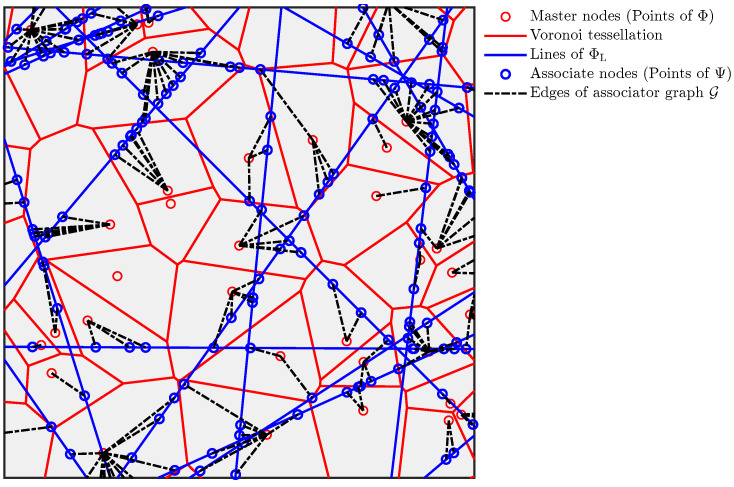
Illustration of a PLP-PPP partitioned by a PV tessellation resulting in a graph G.

Further, each node of the first type (PP Ψ) is connected with one (and only one) node of the second type (PP Φ). Hence, we call the nodes of the first type (PP Ψ) as *the associate nodes* and the second (PP Φ) as *the master nodes*. Further, the association is based on the mutual distance, where each associate node is connected to the closest master node. This association is commonly used in selecting access points in wireless networks or fusion nodes in sensor networks in order to maximize network performance. In general, in any scenario where the quality of an interaction between two nodes decreases with the distance between them, this association would be practically relevant. This association results in a simple graph G with edges from the points of Ψ to Φ where each association is represented as an edge. The scenario is illustrated in Figure 2. As mentioned earlier, we term this graph the *random bipartite geometric associator graph*.

The above association can also be visualized in terms of Voronoi partitions. In order to understand that, consider the space partitioned by the Voronoi tessellation V generated by the master nodes Φ. In V, each master node is associated with a Voronoi cell (region). In particular, the Voronoi cell associated with the typical master node is termed as the *typical cell*. The Voronoi cell of a node located at y, $C_{y}$ is defined as

$$C_{y}=\{x\in R^{2}:y=\arg\min_{y_{i}\in \Phi }∥x-y_{i}∥\}.$$

This Poisson Voronoi (PV) tessellation V partitions the PLP-PPP Ψ into smaller PPs $\left\{\Psi_{y}\right\}$ associated with master nodes {y} where $\Psi_{y}$ is comprised of points of Ψ falling inside Voronoi cell $C_{y}$. It is clear from this construction that $\Psi_{y}$ is the same as the set of the associate nodes connected with the master node y in graph G. Let $S_{p}$ denote the number of edges associated with the typical master node of Φ, which essentially denotes its *node degree*. It is the same as the number of points of Ψ falling in the typical cell of V which can also be understood as the *load* on the typical cell in many applications (such as wireless cellular networks).

Note that the points in $\Psi_{y}$ are located at the chords formed by $\Phi_{L}$ in $C_{y}$. This brings us to the notion of the typical chord of the typical Voronoi cell which is defined as any randomly drawn chord in the typical Voronoi cell without any selection bias.

For this setup, we are interested in deriving the distribution of the node degree along with various statistics including its mean and variance. In order to do that, we need several intermediate results, which is derived in the next section.

## 3. Cell Perimeter, Area and Chord Distribution under PV Tessellation

In this section, we will present some important expressions including the PDF of the area, and perimeter of the Voronoi cell, and the chord length distribution in the Voronoi cell, which will be useful in the subsequent sections of this paper.

### 3.1. Area, Perimeter, and Chord Length Distribution of the Typical Cell

The analytical distributions for the area and perimeter for the typical cell defined above are presented in [23]. Since the analytical expressions are not in closed form and hence unwieldy to work with, we will instead use the empirical PDFs of area and perimeter presented in [24]. Let the Voronoi region associated with the typical cell be denoted by $V_{t}$ with area $|V_{t}|$ and perimeter *Z*. The empirical PDFs of $|V_{t}|$ and *Z* are given as

(7) $$\begin{array}{cc} \; & f_{|V_{t}|}\left(v_{t}\right)=\lambda g\left(1.07950,3.03226,3.31122,\lambda v_{t}\right), \end{array}$$

(8) $$\begin{array}{cc} \; & f_{Z}\left(z\right)=\sqrt{\lambda }\frac{1}{4}g\left(2.33609,2.97006,7.58060,\sqrt{\lambda }\frac{z}{4}\right), \end{array}$$

where g(·) denotes the generalized Gamma distribution presented in (2). Further, $E[V_{t}]=1/\lambda$, $E\left[Z\right]=4/\sqrt{\lambda }$.

Since the points of Ψ lie on the lines, we need to understand the statistics of the chords formed by the intersection of the PLP $\Phi_{L}$ and the typical cell $V_{t}$. For that, we focus on the typical chord and provide useful results related to this typical chord length. The PDF $f_{C}\left(c\right)$ of the length *C* of the typical chord is [25] $f_{C}\left(c\right)=$

(9) $$\begin{array}{cc} \; & =\left(\pi /2\right)\lambda^{\frac{3}{2}}\int_{0}^{\pi }\int_{0}^{\infty }\left[\lambda {\left(V^{\left(1\right)}\left(c,y,r\left(c,\theta \right)\right)\right)}^{2}-V^{\left(2\right)}\left(c,y,r\left(c,\theta \right)\right)\right]e^{-\lambda V(c,y,r(c,\theta ))}ydyd\theta , \end{array}$$

where V(c,y,r(c,θ)) is the area of union of two disk of radius *y* and r(c,θ) with centers *c* distance away, $V^{\left(k\right)}\left(\;\cdot \;\right)$ denotes the *k*-th derivative of V(·) with respect to *c* and $r\left(c,\theta \right)=\sqrt{y^{2}+c^{2}-2yc\cos\theta }$. Further, $E\left[C\right]=\pi /\left(4\sqrt{\lambda }\right)$ [25]. For the completeness of (9), we now present the V(·) and its first and second derivatives with respect to *c* in the following lemma.

**Lemma** **1.** *The area of the union of two disks of radius y and $r\left(c,\theta \right)\left(=\sqrt{y^{2}+c^{2}-2yc\cos\theta }\right)$ with centers c distance away is*

$$\begin{array}{cc} \; & V\left(c,y,r\left(c,\theta \right)\right)=2\pi y^{2}-2\pi yc\cos\theta -y^{2}\left(\theta -0.5\sin(2\theta )\right)+\pi c^{2}\\ \; & -\left(y^{2}-2yc\cos\theta +c^{2}\right)\left(\alpha \left(c\right)-0.5\sin2\alpha \left(c\right)\right), \end{array}$$

*where $\alpha \left(c\right)=\cos^{-1}\left(\frac{c-y\cos\theta }{r(c,\theta )}\right)$, further*

$$\begin{array}{cc} \; & \frac{\partial V(c,y,r(c,\theta ))}{\partial c}=V^{\left(1\right)}\left(c,y,r\left(c,\theta \right)\right)=2\left(c-y\cos\theta \right)\left(\pi -\alpha \left(c\right)\right)+2y\sin\theta .\\ \; & \frac{\partial^{2}V\left(c,y,r\left(c,\theta \right)\right)}{\partial c^{2}}=V^{\left(2\right)}\left(c,y,r\left(c,\theta \right)\right)=\frac{2y\sin\theta \cos(\alpha (c))}{r(c,\theta )}+2\cos^{-1}\left(\frac{y\cos\theta -c}{r(c,\theta )}\right). \end{array}$$

We also provide a result about the dependence of the chord length of a convex polygon on the perimeter of the polygon in the following propositions. For the proofs, please refer to Appendix A and Appendix B.

**Proposition** **1.** *The length of any chord in a convex polygon is upper bounded by half of its perimeter.*

**Proposition** **2.** *The lengths of random chords of a convex polygon conditioned on the perimeter of the polygon are dependent RVs.*

### 3.2. Area, Perimeter, and Chord Length Distribution of the Zero-Cell

We now derive the same set of results for the zero-cell, which is defined as the Voronoi cell that contains the typical point of Ψ. Due to the stationarity of Ψ, we can assume that the typical point is located at the origin. Mathematically, the zero-cell $C_{o}$ can be written as

$$C_{o}=\left\{x\in R^{2}:\arg\min_{y_{i}\in \Phi }∥x-y_{i}∥=\arg\min_{y_{i}\in \Phi }∥y_{i}∥\right\}.$$

Here, the expression $\arg\min_{y_{i}\in \Phi }∥x-y_{i}∥$ represents the master node that serves the associate node located at x. If this master node is the same as the master node serving the origin, i.e., $\arg\min_{y_{i}\in \Phi }∥y_{i}∥$, it indicates that the location x falls within the serving region of the master node closest to the origin, or in other words, it falls in the zero cell. Therefore, all locations x satisfying the above condition constitute the zero cell. The master node with which the zero-cell is associated with is termed *the tagged master node*. The area distribution of the zero-cell can be obtained using the area bias sampling [1] and can be expressed as

$$\begin{array}{c} f_{|V_{t_{o}}|}\left(v_{t_{o}}\right)=\frac{v_{t_{o}}f_{|V_{t}|}\left(v_{t_{o}}\right)}{E[|V_{t}|]}=\lambda v_{t_{o}}f_{|V_{t}|}\left(v_{t_{o}}\right)=\lambda g\left(1.07950,3.03226,4.31122,\lambda v_{t_{o}}\right), \end{array}$$

where note that $\frac{b^{c/a}}{\Gamma (c/a)}=\frac{b^{(c+1)/a}}{\Gamma ((c+1)/a)}$ for a=1.07950, b=3.03226, c=3.31122. Now, we present the empirical PDF of the zero-cell’s perimeter. Inspired by the well-accepted empirical distributions presented in the literature to find the PDF of the area perimeter of typical cell [24], we have also used a three-variable generalized gamma distribution to fit the distribution of perimeter of the zero-cell of PVT. These empirical approximations are common (and necessary) while dealing with Voronoi tessellations. Using simulations, we generate $10^{5}$ samples of zero-cells and compute the empirical PDF of the zero-cell’s perimeter. We fit a generalized Gamma distribution’s PDF (2) via maximum likelihood estimation (MLE) to determine the parameters (a,b, and *c*). Consequently, the fitted PDF of the zero-cell’s perimeter is

(10) $$\begin{array}{c} f_{Z^{\prime }}\left(z^{\prime }\right)=\sqrt{\lambda }g\left(2.1804,0.16839,10.2823,\sqrt{\lambda }z^{\prime }\right), \end{array}$$

where g(·) is given in (2). Further, $E\left[Z^{\prime }\right]=\frac{4.4906}{\sqrt{\lambda }}$.

**Figure 3:**
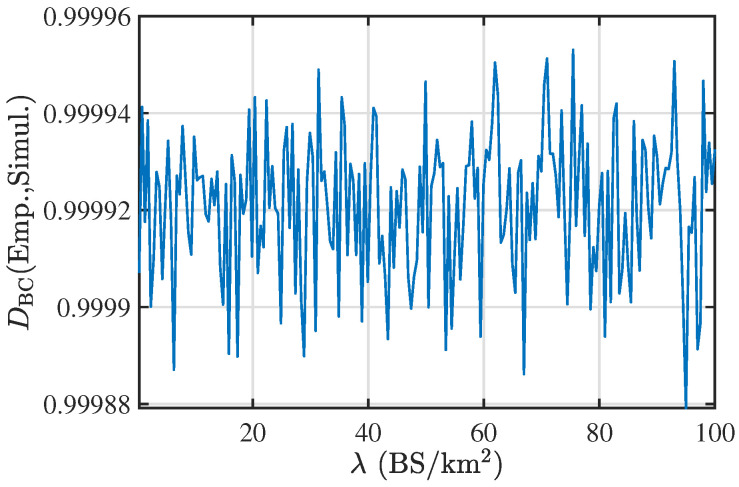
The plot showing the BC coefficient between the empirical (Emp.) PDF and the PDF obtained from simulation (Simul.) of the perimeter of the zero-cell. The plot demonstrates a high accuracy of our result for a range of values of λ.

To quantify the accuracy of the PDF presented in (10), we plot the Bhattacharya’s coefficient (BC) [26] between the PDF given in (10) and the empirical PDF obtained using simulations. The BC coefficient measures the similarity between the two PDFs of continuous RV or two PMFs of discrete RV. Let the PDFs and PMFs for continuous and discrete probability distributions are defined as p(x),q(x),p(ω) and q(ω), respectively, then the $D_{\mathrm{BC}}$ between the continuous PDFs and discrete PMFs is

(11) $$\begin{array}{c} D_{\mathrm{BC}}\left(p,q\right)=\int \sqrt{p(x)q(x)}dx,\;\;D_{\mathrm{BC}}\left(p,q\right)=\sum_{\omega \in \Omega }\sqrt{p(\omega )q(\omega )}, \end{array}$$

respectively. Here, we would like to highlight that the BC ranges from 0 to 1, and a value close to 1 denotes the higher similarity between the distributions. Figure 3 presents the BC coefficient between the fitted and the empirical PDF of the perimeter of the zero-cell. As the BC coefficient is close to 1, the derived PDF is close to the exact PDF of the perimeter of the zero-cell.

For the chord distribution of the zero-cell, we can easily identify that there are two types of chords for the zero-cell (i) the *tagged chord* that goes through the typical point (in our case, the origin) with its length denoted as $C_{o}$, and (ii) the randomly selected chord of zero-cell with length $\tilde{C}$. The length distribution of $C_{o}$ is presented in [20] as

(12) $$\begin{array}{c} f_{C_{o}}\left(c_{o}\right)=\int_{0}^{c_{o}}f_{L_{1},L_{2}}\left(c_{o},c_{o}-l_{2}\right)dl_{2}, \end{array}$$

where

(13) $$\begin{array}{c} f_{L_{1},L_{2}}\left(l_{1},l_{2}\right)=8\lambda^{3}\int_{0}^{\pi }\int_{0}^{\infty }e^{-\lambda V(l_{1}+l_{2},r\left(l_{1}\right),r\left(l_{2}\right))}v_{1}^{\left(1\right)}\left(l_{1}\right)v^{\left(2\right)}\left(l_{2}\right)ydyd\theta , \end{array}$$

where V(·) is given in Lemma 1 and

$$\begin{array}{c} \;v_{1}\left(l_{1}\right)\;=\;2\left(l_{1}+y\cos\theta \right)\left(\pi -\alpha_{1}\left(l_{1}\right)\right)\;+\;2y\sin\theta ,\;\;v_{2}\left(l_{2}\right)\;=\;2\left(l_{2}+y\cos\theta \right)\left(\pi -\alpha_{2}\left(l_{2}\right)\right)\;+\;2y\sin\theta \end{array}$$

and $\alpha_{1}\left(l_{1}\right)=\cos^{-1}\left(\frac{l_{1}-y\cos\theta }{r(l_{1})}\right)$ and $\alpha_{2}\left(l_{2}\right)=\cos^{-1}\left(\frac{l_{2}+y\cos\theta }{r(l_{2})}\right)$. We now give the approximate length distribution of $\tilde{C}$ in the following Lemma. Please see Appendix C for the proof.

**Lemma** **2.** *The length distribution of $\tilde{C}$ of any randomly selected chord is approximately given as $f_{\tilde{C}}\left(\tilde{c}\right)\approx \eta f_{C}\left(\eta \tilde{c}\right)$ with η=0.8769.*

In the next section, we present the distance distributions for PLP-PPP which are not only useful on their own right but will also play a role in our subsequent analysis.

## 4. Distance Distributions for PLP-PPP

In this section, we present the PDFs and CDFs of the *k*-th CD and NND for the PLP-PPP.

### 4.1. Distribution of the *k*-th CD

The *k*-th CD is defined as the distance of the *k*-th point of Ψ from an arbitrary point in $R^{2}$. Let S(r) denote the number of points of Ψ falling in $b_{2}\left(o,r\right)$. From the definition of *k*-th CD [27], the CDF $F_{R_{k}}\left(r\right)$ is

$$\begin{array}{c} F_{R_{k}}\left(r\right)=1-\sum_{m=0}^{k-1}P\left[S(r)=m\right]. \end{array}$$

Hence, we first derive the PGF followed by the PMF for S(r), i.e., the number of points of Ψ falling in ball $b_{2}\left(o,r\right)$. Note that an alternative formula for the PMF of S(r) is presented in [10] (Lemma 4.4) which is slightly more complicated compared to the one presented here. Please see Appendix D for the proof of the following result.

**Theorem** **1.** *The PGF for S(r) is*

(14) $$\begin{array}{cc} \; & P_{S(r)}\left(s\right)=\exp\left(g_{p}\left(s\right)\right), \end{array}$$

*where*

$$\begin{array}{c} g_{p}\left(s\right)=2\pi \lambda_{L}\left(\int_{0}^{r}\exp\left(2\lambda_{R}\sqrt{r^{2}-\rho^{2}}\left(s-1\right)\right)d\rho -r\right). \end{array}$$

Let $h_{m}\left(r\right)=g_{p}^{\left(m\right)}\left(0\right)/m!$ denote the *m*-th derivative of $g_{p}\left(s\right)$ at s=0 and $h_{0}\left(r\right)=g_{p}\left(0\right)$. In the next result, we derive the PMF of S(r) using (3).

**Corollary** **1.** *The PMF of S(r) is*

(15) $$\begin{array}{cc} \; & P\left[S(r)=m\right]=\exp\left(h_{0}\left(r\right)\right)\sum_{N_{m}}\frac{{\left(h_{1}\left(r\right)\right)}^{n_{1}}\cdots {\left(h_{m}\left(r\right)\right)}^{n_{m}}}{n_{1}!\cdots n_{m}!}, \end{array}$$

*where the sum is defined in (3) and $h_{m}\left(r\right)$ is given as*

(16) $$\begin{array}{cc} h_{m}\left(r\right) & =\frac{g_{p}^{\left(m\right)}\left(0\right)}{m!}=\frac{2\pi \lambda_{L}}{m!}\left(\int_{0}^{r}{\left(2\lambda_{R}\sqrt{r^{2}-\rho^{2}}\right)}^{m}\exp\left(-2\lambda_{R}\sqrt{r^{2}-\rho^{2}}\right)d\rho \right), \end{array}$$

(17) $$\begin{array}{cc} \; & =\frac{2\pi \lambda_{L}}{m!}\int_{0}^{\pi /2}{\left(2\lambda_{R}\cos\theta \right)}^{m}r^{m+1}\exp\left(-2\lambda_{R}r\cos\theta \right)\cos\theta d\theta . \end{array}$$

The last expression of the above Corollary is obtained by substituting ρ=rsinθ. From Theorem 1, we obtain the distribution of *k*-th CD as given below.

**Corollary** **2.** *The CDF of k-th CD for* Ψ *is*

(18) $$\begin{array}{c} F_{R_{k}}\left(r\right)=1-\exp\left(h_{0}\left(r\right)\right)\sum_{m=0}^{k-1}\sum_{N_{m}}\frac{{\left(h_{1}\left(r\right)\right)}^{n_{1}}\cdots {\left(h_{m}\left(r\right)\right)}^{n_{m}}}{n_{1}!\cdots n_{m}!}. \end{array}$$

Now, before deriving the CDFs and PDFs for some special cases such as k=1 and 2, we derive the first derivative of $h_{m}\left(r\right)$ denoted by $h_{m}^{\left(1\right)}\left(r\right)$ with respect to *r* which is crucial for computing the PDF of CD.

$$\begin{array}{cc} h_{m}^{\left(1\right)}\left(r\right) & =\frac{2\pi \lambda_{L}}{m!}\int_{0}^{\pi /2}{\left(2\lambda_{R}\cos\theta \right)}^{m}\left(\left(m+1\right)r^{m}e^{-2\lambda_{R}r\cos\theta }\right. \end{array}$$

(19) $$\begin{array}{cc} \; & \left.-2\lambda_{R}\cos\theta r^{m+1}e^{-2\lambda_{R}r\cos\theta }\right)\cos\theta d\theta , \end{array}$$

(20) $$\begin{array}{cc} h_{0}^{\left(1\right)}\left(r\right) & =h_{1}^{\left(1\right)}\left(r\right)-1. \end{array}$$

**Corollary** **3.** *The CDF and PDF of CD for k=1 and* 2 *are*

$$\begin{array}{cc} \; & F_{R_{1}}\left(r\right)=1-e^{h_{0}\left(r\right)},\;\;\;\;f_{R_{1}}\left(r\right)=\exp\left(h_{0}\left(r\right)\right)\left(1-h_{1}^{\left(1\right)}\left(r\right)\right),\\ \; & F_{R_{2}}\left(r\right)=1-e^{h_{0}\left(r\right)}\left(1+h_{1}\left(r\right)\right),\;\;f_{R_{2}}\left(r\right)=f_{R_{1}}\left(r\right)\left(1+h_{1}\left(r\right)\right)-e^{h_{0}\left(r\right)}h_{1}^{\left(1\right)}\left(r\right). \end{array}$$

### 4.2. Distribution of the k-th NND

Along the same lines as the *k*-th CD, the *k*-th NND is defined as the distance of *k*-th nearest point from the typical point of Ψ. Without loss of generality, we assume that the typical point is located at the origin, hence o∈Ψ. Let M(r) denote the number of points of Ψ∖{o} falling in $b_{2}\left(o,r\right)$. From the definition [27], CDF of *k*-th NND $R_{k}^{{}^{\prime }}$ is

$$\begin{array}{c} F_{R_{k}^{{}^{\prime }}}\left(r\right)=1-\sum_{m=0}^{n-1}P\left[M(r)=m|o\in \Psi \right]=1-\sum_{m=0}^{n-1}P^{o!}\left[M(r)=m\right], \end{array}$$

where $P^{o!}\left[\;\cdot \;\right]$ denotes the probability under the reduced Palm version of Ψ. Before deriving the CDF of *k*-th NND, we first derive the PGF of M(r). For the proof of the following results please refer to Appendix E.

**Theorem** **2.** *The PGF of M(r) is*

(21) $$\begin{array}{c} P_{M(r)}\left(s\right)=e^{\lambda_{R}2r\left(s-1\right)}\exp\left(g_{p}\left(s\right)\right). \end{array}$$

**Corollary** **4.** *The PMF of M(r) is*

(22) PM(r)=m=∑j=0mmj(2λRr)m−jexph0(r)∑Nj(h1(r))n1⋯(hj(r))njn1!⋯nj!.

Using the PMF of M(r), we may derive the CDFs and PDFs of *k*-th NND as

**Corollary** **5.** *The CDF $F_{R_{k}^{{}^{\prime }}}\left(r\right)$ for k-th NND of* Ψ *is*

(23) FRk′(r)=1−∑m=0k−1∑j=0mmj(2λRr)m−je−2λRrexph0(r)∑Nj(h1(r))n1⋯(hj(r))njn1!⋯nj!,

*where $h_{j}\left(r\right)$ is provided in (16).*

**Corollary** **6.** *The CDF and PDF of NND for k=1,2 is*

$$\begin{array}{cc} \; & F_{R_{1}^{{}^{\prime }}}\left(r\right)=1-e^{h_{0}\left(r\right)}e^{-2\lambda_{R}r},\;\;f_{R_{1}^{{}^{\prime }}}\left(r\right)=e^{h_{0}\left(r\right)-2\lambda_{R}r}\left(2\lambda_{R}-h_{0}^{\left(1\right)}\left(r\right)\right),\\ \; & F_{R_{2}^{{}^{\prime }}}\left(r\right)=1-\exp\left(h_{0}\left(r\right)\right)e^{-2\lambda_{R}r}\left(1+2\lambda_{R}r+h_{1}\left(r\right)\right),\\ \; & f_{R_{2}^{{}^{\prime }}}\left(r\right)=f_{R_{1}^{{}^{\prime }}}\left(r\right)\left(1+2\lambda_{R}r+h_{1}\left(r\right)\right)-e^{h_{0}\left(r\right)-2\lambda_{R}r}\left(2\lambda_{R}+h_{1}^{\left(1\right)}\left(r\right)\right), \end{array}$$

*where $h_{j}\left(r\right)$ can be obtained from (19).*

The approach that we utilized to determine the distance distribution for PLP-PPP is rather general and has several uses in a vehicular network modeled as a PLP-PPP, such as deriving the load on the BSs which we will revisit in subsequent sections.

## 5. Node Degree Distribution for the Typical Master Node in the Associator Graph G

We now derive the distribution of the node degree of the typical master node in graph G which is equal to the number $S_{p}$ of points falling in the typical Voronoi cell of Φ. For the exact analysis, one can adopt the following approach.

S–1 Conditioned on the perimeter *Z* of the typical Voronoi cell, using the property of PLP, the number of lines *n* intersecting the Voronoi cell is Poisson RV with mean $\lambda_{L}Z$. The empirical distribution of *Z* is provided in [24] and stated above in (8).
S–2 Conditioned on the perimeter *Z* and *n*, we compute the length distribution (PDF) of all chords of the typical Voronoi cell.
S–3 Once the PDF of the sum of lengths of the *n* chords is obtained in S-2, we can decondition it using the distribution of *n* conditioned on *Z*. Finally, using the distribution of *Z*, we obtain the PGF for the node degree of the typical master node.

Note that Proposition 1 states that the chord length depends on the perimeter while Proposition 2 states that conditioned on the perimeter *Z*, the lengths of the chords are not independent, which requires us to derive the joint PDF. Since the joint distribution of chord lengths conditioned on *Z* is not available, the exact analysis is difficult. Therefore, we present two methods of approximating the node degree for the typical Voronoi cell below.

### 5.1. Approximation-1-typical (App1−typ) Approach

In the App1−typ approach to obtain the approximate value ${\hat{S}}_{p}$, we make the following approximation in the S-2 step above. Conditioned on the perimeter *Z* and *n*, we assume that the chord length distribution of the typical Voronoi cell is independent of the perimeter, i.e., $f_{C|Z}\left(c\right)=f_{C}\left(c\right)$ and conditioned on the perimeter *Z*, the lengths of the chords are independent of each other, i.e., $f_{C_{1},C_{2},\ldots C_{n}|Z}\left(c_{1},c_{2},\ldots ,c_{n}\right)=f_{C_{1}}\left(c_{1}\right)f_{C_{2}}\left(c_{2}\right)\ldots f_{C_{n}}\left(c_{n}\right)$. Due to these two assumptions, the PGF of ${\hat{S}}_{p}$ is the product of the PGF’s of the number of points on each chord inside the typical Voronoi cell. The following theorem provides the PGF of ${\hat{S}}_{p}$ along with its PMF and key moments. Please see Appendix F for the proof.

**Theorem** **3.** *(App1−typ) The PGF $P_{{\hat{S}}_{p}}\left(s\right)$ for the approximated node degree on the typical Voronoi cell is*

(24) $$\begin{array}{cc} P_{{\hat{S}}_{p}}\left(s\right) & =\int_{z=0}^{\infty }\exp\left(-\lambda_{L}z\left(1-\int_{0}^{\infty }\exp\left(\lambda_{R}c\left(s-1\right)\right)f_{C}\left(c\right)dc\right)\right)f_{Z}\left(z\right)dz. \end{array}$$

*The PMF of ${\hat{S}}_{p}^{}$ is*

(25) $$\begin{array}{cc} \; & P\left[{\hat{S}}_{p}^{}=k\right]=\int_{0}^{\infty }\exp\left(h_{p}\left(0,z\right)\right)\sum_{N_{k}}\frac{{\left(h_{p,1}\left(z\right)\right)}^{n_{1}}\cdots {\left(h_{p,k}\left(z\right)\right)}^{n_{k}}}{n_{1}!\cdots n_{k}!}f_{Z}\left(z\right)dz, \end{array}$$

*where $h_{p}\left(s,z\right)$ and $h_{p,k}\left(z\right)$ are*

(26) $$\begin{array}{cc} \; & h_{p}\left(s,z\right)=\lambda_{L}z\left(\int_{0}^{\infty }e^{\lambda_{R}c\left(s-1\right)}f_{C}\left(c\right)dc-1\right),\;h_{p,k}\left(z\right)=\left(\frac{\lambda_{L}z}{k!}\right)\int_{0}^{\infty }{\left(\lambda_{R}c\right)}^{k}e^{-\lambda c}f_{C}\left(c\right)dc. \end{array}$$

*Further, the mean and the variance of ${\hat{S}}_{p}$ are*

(27) $$\begin{array}{cc} \; & E\left[{\hat{S}}_{p}\right]=\frac{\mu }{\pi }E\left[Z\right]E\left[C\right]=\mu /\lambda , \end{array}$$

(28) $$\begin{array}{cc} \; & \mathrm{Var}\left[{\hat{S}}_{p}\right]={\left(\mu /\pi \right)}^{2}E\left[Z^{2}\right]{\left(E[C]\right)}^{2}+\left(\mu /\pi \right)\lambda_{R}E\left[C^{2}\right]E\left[Z\right]+\mu /\lambda -{\left(\mu /\lambda \right)}^{2}. \end{array}$$

A similar approach to finding the approximate distribution in such models has been used in [19,20]. In particular, in [19] authors first derive the Laplace functional (LF) of sum *W* of the length of all chords falling inside the typical Voronoi cell conditioned on the perimeter *Z*. In [20] authors assumed that the chord lengths in the typical Voronoi cell are independent RVs and then derived the load distribution for the Cox process driven by PLP by superimposing the 1D MCP on each line of PLP. It is important to note that in both papers, the lengths of these chords conditioned on the perimeter are assumed to be independent RVs which is an approximation as shown in the Propositions 1 and 2. Then, using the PGFL of PLP and deconditioning with the perimeter *Z*’s distribution, the PMF of the load is obtained in terms of the derivative of LF. Here, we would like to highlight that the PGFs and corresponding PMFs of the node degree presented in this paper are simpler as compared to [19]. For example, in [19] the PMF of load involves a higher-order derivative of a function; however, the PMF presented in the paper is in closed form. Due to the simple and closed-form nature of the PMFs, the numerical implementation of PMFs is much faster in this case. Moreover, we are able to obtain the mean and variance of the ode degree easily from the PGF, which may not always be as easy from the PMF. Another significant advantage of the proposed techniques for deriving the node degree distribution in both approximations is that we primarily utilized the two PGFLs, namely the PGFL of PLP and PPP. An important consequence of this observation is that these approximations can be easily extended to the variants of the model used in this paper in which the PP used to describe the placement of points on lines does not have to be a PPP as long as it has a known PGFL. Therefore, it is possible to extend these results to the settings in which vehicles exhibit clustering or repulsion as long as the PP used to describe these placements has a known PGFL.

### 5.2. Approximation-2-typical (App2−typ) Approach

In this approach, $S_{p}$ is approximated by ${\tilde{S}}_{p}$, which is the number of points in the ball of area equal to the area $|V_{t}|$ of the typical Voronoi cell. Hence, the radius of the ball is a random variable given as $R_{t}=\sqrt{|V_{t}|/\pi }$ with the distribution

(29) $$\begin{array}{c} f_{R_{t}}\left(r_{t}\right)=2\pi r_{t}f_{|V_{t}|}\left(\pi r_{t}^{2}\right)=\sqrt{\lambda }g\left(2.15900,3.03226\pi^{1.07950},6.62244,\sqrt{\lambda }r_{t}\right). \end{array}$$

Here, $f_{|V_{t}|}\left(\;\cdot \;\right)$ is given in (7). Therefore, ${\tilde{S}}_{p}=S\left(R_{t}\right)$ where S(·) is given in Theorem 1. The PGF and corresponding PMF of the approximate node degree ${\tilde{S}}_{p}$ are given below.

**Theorem** **4.** *(App2−typ) The PGF and PMF for ${\tilde{S}}_{p}$ are*

(30) $$\begin{array}{c} P_{{\tilde{S}}_{p}}\left(s\right)=\int_{r_{t}=0}^{\infty }P_{S(r_{t})}\left(s\right)f_{R_{t}}\left(r_{t}\right)dr_{t},\;\;P\left({\tilde{S}}_{p}=m\right)=\int_{r_{t}=0}^{\infty }P\left(S(r_{t})=m\right)f_{R_{t}}\left(r_{t}\right)dr_{t}, \end{array}$$

*where PGF and PMF of S(r) is given in Theorem 1.*

Using the properties of PGF stated in (1), we can compute the mean and the variance of the approximate node degree as given in the following. Please see Appendix G for the proof.

**Corollary** **7.** *The mean and the variance of ${\tilde{S}}_{p}$ are*

(31) $$\begin{array}{cc} \; & E\left[{\tilde{S}}_{p}\right]=\mu \pi E\left[R_{t}^{2}\right]=\mu /\lambda , \end{array}$$

(32) $$\begin{array}{cc} \; & \mathrm{Var}\left[{\tilde{S}}_{p}\right]={\left(\pi \mu \right)}^{2}E\left[r_{t}^{4}\right]+\left(16/3\right)\lambda_{R}\mu E\left[r_{t}^{3}\right]+\mu /\lambda -{\left(\mu /\lambda \right)}^{2}, \end{array}$$

*where $E\left[R_{t}^{2}\right]=\frac{1}{\left(\pi \lambda \right)}$, $E\left[R_{t}^{3}\right]=\frac{0.198}{\lambda^{3/2}}$, and $E\left[r_{t}^{4}\right]=\frac{0.130}{\lambda^{2}}$.*

It can be seen that the expressions obtained via App2−typ are simpler than the App1−typ. We will numerically compare the accuracy of the two approaches in Section 9.2.

## 6. Node Degree Distribution for the Tagged Master Node in the Associator Graph G

We now derive the distribution of node degree of the master node of the zero-cell in graph G which is equal to the number $M_{p}$ of points falling in the zero-cell. It can also be seen as the load on the zero cell. Just like the approach discussed in the previous section for the typical cell, one can adopt the following approach for this derivation.

S–1 As soon as we condition on the typical point of Ψ, we know that there is a line passing through that location (since points of Ψ lie on the lines). Recall that the chord corresponding to this line has been defined as the tagged chord above. In addition, there are other chords in the zero-cell, on which points are located. Hence, the node degree $M_{p}$ is the sum of the number of points on the tagged chord and the other chords of zero-cell.
S–2 Hence, the PGF for $M_{p}$ is the product of the PGF of the number of points falling on the tagged chord and the PGF for the number of points falling on the rest of the chords of the zero-cell.
S–3 The length distribution of the tagged chord is presented in (12). Using that, we can compute the PGF of the number of points falling on the tagged chord.
S–4 Conditioned on the perimeter of the zero-cell, the number of other chords is Poisson distributed. Using the distribution of the sum of their lengths conditioned on the perimeter of the zero-cell and their number, the PGF of the number of points on them can be computed which can be further averaged over the two.

As we discussed in the case of the typical cell in the previous section, the exact analysis is intractable because of the lack of joint distribution of the chord lengths. In order to overcome this challenge, we present two approximations similar to the typical cell case.

### 6.1. Approximation-1-zero (App1−zero)

In this approach, we approximate $M_{p}$ by ${\hat{M}}_{p}$ by assuming that the length of the other chords (other than the tagged chord) in the zero-cell are independent RVs whose distributions do not depend on the perimeter. Further, owing to symmetry, these lengths are identically distributed. The length distribution of each chord can then be given by the distribution $f_{\tilde{C}}\left(c\right)$ of the typical chord length $\tilde{C}$ of the zero-cell. We derive this distribution conditioned on the zero-cell perimeter. Averaging over the distribution of the perimeter, we obtain the following result. Please see Appendix H for the proof.

**Theorem** **5.** *The PGF of the approximate node degree ${\hat{M}}_{p}$ on the zero-cell under the App1−zero is*

(33) $$\begin{array}{cc} P_{{\hat{M}}_{p}}\left(s\right)= & \int_{z^{{}^{\prime }}=0}^{\infty }\exp\left(-\lambda_{L}z^{{}^{\prime }}\left(1-\int_{0}^{\infty }\exp\left(\lambda_{R}\tilde{c}\left(s-1\right)\right)f_{\tilde{C}}\left(\tilde{c}\right)dc\right)\right)f_{Z^{\prime }}\left(z^{\prime }\right)dz^{\prime }\\ \; & \;\times \int_{c_{o}=0}^{\infty }e^{\lambda_{R}c_{o}\left(s-1\right)}f_{C_{o}}\left(c_{o}\right)dc_{o}, \end{array}$$

*where $f_{\tilde{C}}\left(\tilde{c}\right)$, $f_{Z^{{}^{\prime }}}\left(z^{{}^{\prime }}\right)$ and $f_{C_{o}}\left(c_{o}\right)$ and are given in Corollary 2, (10), and (12), respectively. Further, the PMF is*

(34) PM^p=m=∑k=0mmk∫co=0∞(λRco)m−kfCo(co)dco×∫0∞exphm(0,z′)∑Nk(hm,1(z′))n1⋯(hm,k(z′))nkn1!⋯nk!fZ′(z′)dz′,

*where $h_{m}\left(s,z^{\prime }\right)$ and $h_{m,k}\left(z^{\prime }\right)$ are*

(35) $$\begin{array}{cc} \; & h_{m}\left(s,z^{\prime }\right)\;=\;\lambda_{L}z^{\prime }\left(\int_{0}^{\infty }e^{\lambda_{R}c\left(s-1\right)}f_{\tilde{C}}\left(\tilde{c}\right)d\tilde{c}-1\right),\;h_{m,k}\left(z^{\prime }\right)\;=\;\frac{\lambda_{L}z^{\prime }}{k!}\int_{0}^{\infty }{\left(\lambda_{R}c\right)}^{k}e^{-\lambda_{R}\tilde{c}}f_{\tilde{C}}\left(\tilde{c}\right)d\tilde{c}. \end{array}$$

As the PGF of ${\hat{M}}_{p}$ is the product of the PGFs of two independent RVs, the mean of ${\hat{M}}_{p}$ is the summation of the mean number of points on the tagged chord and the other chords of the zero cell. The next corollary presents this result.

**Corollary** **8.** *The mean of node degree on the zero-cell is approximated as*

$$\begin{array}{cc} \; & E\left[{\hat{M}}_{p}\right]=\frac{\mu }{\pi }E\left[\tilde{C}\right]E\left[Z^{\prime }\right]+\frac{4\sqrt{\lambda }\lambda_{R}}{\pi }E\left[C^{2}\right]=\frac{1.28\mu }{\lambda }+\frac{4\sqrt{\lambda }\lambda_{R}}{\pi }E\left[C^{2}\right]. \end{array}$$

Similarly, we can derive the variance of ${\hat{M}}_{p}$.

### 6.2. Approximation-2-zero (App2−zero) Approach

Similar to the typical cell, we can approximate the zero-cell $V_{t_{o}}$ with a ball $b_{2}\left(y,R_{o}\right)$ of equal area. Here, y is the location of the master node corresponding to $V_{t_{o}}$. For equal area $|V_{t_{o}}|=\pi R_{o}^{2}$ which gives $R_{o}=\sqrt{|V_{t_{o}}|/\pi }$. Hence, the PDF of $R_{o}$ is

(36) $$\begin{array}{c} f_{R_{o}}\left(r_{o}\right)=2\pi^{2}\lambda r_{o}^{3}f_{|V_{t}|}\left(\pi r_{o}^{2}\right)=\lambda \pi r_{o}^{2}f_{R_{t}}\left(r_{o}\right), \end{array}$$

where $f_{R_{t}}\left(\;\cdot \;\right)$ is given in (29). Under this approximation, the node degree ${\tilde{M}}_{p}$ corresponding to the zero-cell will be equal to the sum of two independent RVs. One RV represents the number of points falling on the tagged chord and the second denotes the number of points in the equivalent ball. This gives us the following PGF. The proof is given in Appendix I.

**Theorem** **6.** *The PGF of ${\tilde{M}}_{p}$ is*

(37) $$\begin{array}{cc} \; & P_{{\tilde{M}}_{p}}\left(s\right)=\int_{r_{o}=0}^{\infty }P_{S(r_{o})}\left(s\right)f_{R_{o}}\left(r_{o}\right)dr_{o}\times \int_{c_{o}=0}^{\infty }e^{\lambda_{R}c_{o}\left(s-1\right)}f_{C_{o}}\left(c_{o}\right)dc_{o}, \end{array}$$

*where the PDFs of $R_{o}$ and $C_{o}$ are given in (36) and (12), respectively. The PMF $P\left[{\tilde{M}}_{p}=m+1\right]$ is*

PM˜p=m+1=∑k=0mmk∫ro=0∞PS(ro)=kfRo(ro)dro×∫co=0∞(λRco)m−kexp−λRcofCo(co)dco,

*where $P(S\left(r_{o}\right)=k)$ is provided in Theorem 1.*

**Corollary** **9.** *The mean of the node degree corresponding to the zero-cell can be approximated as*

$$\begin{array}{cc} \; & E\left[{\tilde{M}}_{p}\right]=\mu \pi E\left[R_{o}^{2}\right]+\lambda_{R}E\left[C_{o}\right]\overset{\left(a\right)}{=}\frac{1.28\mu }{\lambda }+\frac{4\sqrt{\lambda }\lambda_{R}}{\pi }E\left[C^{2}\right], \end{array}$$

*where (a) is due to $E\left[R_{o}^{2}\right]=\frac{1.28}{\pi \lambda }$. The second derivative $P_{{\tilde{M}}_{p}}^{\left(2\right)}\left(s\right)$ of the PGF at s=1 is*

(38) $$\begin{array}{c} {\left[P_{{\tilde{M}}_{p}}^{\left(2\right)}\left(s\right)\right]}_{s=1}={\left(\pi \mu \right)}^{2}E\left[R_{o}^{4}\right]+\frac{16}{3}\lambda_{R}\mu E\left[R_{o}^{3}\right]+\frac{2.48\lambda_{R}\mu }{\lambda }E\left[C_{o}\right]+\lambda_{R}^{2}E\left[C_{o}^{2}\right]. \end{array}$$

*Using the second derivative and (1), we obtain the variance of ${\tilde{M}}_{p}$.*

Similar to $R_{t}$, $E\left[R_{o}^{3}\right]=\frac{0.2805}{\lambda^{3/2}}$ and $E\left[R_{o}^{4}\right]=\frac{0.2017}{\lambda^{3}}$. The approach that we utilized to determine the distributions of distances and node degree for PLP-PPP is rather general and has several applications in wireless applications including load distribution on each BS in a vehicular network modeled as a PLP-PPP.

The rest of this paper is devoted to several applications of the models and results discussed so far to problems of practical interest in wireless networks.

## 7. Application Area 1: Vehicular Communications Networks

**Figure 4:**
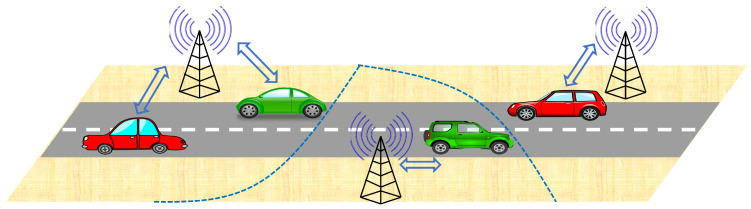
Illustration of a scenario where the vehicles connect with their nearest BSs.

The associator graph studied in this paper is largely inspired by vehicular networks. Therefore, it is quite befitting to consider this as our primary application area. A vehicular communication network consists of multiple vehicles located on a system of roads and a wireless network deployed in the same space to provide connectivity to these vehicles (Figure 4). Because of its ubiquity, we will assume this wireless network to be a cellular network that provides cellular connectivity to the vehicular nodes. As discussed next, this setup can be directly mapped to the model introduced in this paper. This will allow us to use the mathematical properties of the model explored in this paper to study several practical aspects of vehicular communications networks.

1. The spatial layout of roads is modeled a PLP $\Phi_{L}=\left\{l_{i}\left(\rho_{i},\phi_{i}\right),\;\forall i\in N\right\}$ with density $\lambda_{L}$.
2. We model the vehicles on the *i*-th road $l_{i}\left(\rho_{i},\phi_{i}\right)$ by an independent PPP $\psi_{i}=\left\{x_{j,i},\forall \left\{j,i\right\}\in N\right\}$ with density $\lambda_{R}$. Therefore, the union of all the vehicles located on each road of $\Phi_{L}$ forms PLP-PPP.
3. The BS locations are modeled as a 2-D PPP $\Phi \equiv \{y_{i}\}$ with density λ. The role of the BS is to provide infrastructure connectivity (V2I) to the vehicles.
4. Further, we assume that the association of a vehicle to its serving BS is based on the maximum average received power. Assuming the same transmit power for all the BSs, this is the same as associating each vehicle with its closest BS. In other words, each vehicle will connect to the BS whose Voronoi cell it falls in. Therefore, if we consider a PV tessellation with the BSs as master nodes, each Voronoi cell will denote the service region of the respective BS.

Owing to their tractability and accuracy, such stochastic geometry based models have gained popularity within the context of vehicular networks. For instance, the authors in [28] have illustrated that these models are, at a minimum, as precise as the grid-based models utilized for wireless standardization by 3GPP. This provides a robust rationale for their incorporation, especially in foundational theoretical studies, such as the one described in this paper.

Note, further that since the vehicular users are confined on the road, it is important to incorporate the impact of roads in the modeling of vehicular users. Hence, the PLP-PPP turns out to be a suitable choice as it accurately captures the randomness in the both components. Modeling these users as PPP may lead to simpler expressions; however, it may not be accurate for a general case as will be seen in the numerical section. Now we construct an associator graph G with BSs as master nodes and vehicles as associate nodes where the association represents the BS each vehicle connects with.

### 7.1. The Load on the Typical BS

For the setting described above, the load served by the typical BS of Φ is defined as the number of vehicles that are connected to it at any given time. Due to the association law discussed above, these are the vehicles falling inside the typical Voronoi cell. Since the resources at the BSs will be shared by these vehicles, it is important to understand the distribution of load. Owing to the stationarity of this setup, the typical point can be placed at the origin. Therefore, the aforementioned load is exactly the node degree of the typical master node in the graph G or the load (i.e., the number of points of Ψ) $S_{p}$ on the typical Voronoi cell as studied in the paper. Hence, we denote this load also by the RV $S_{p}$. Its two approximate distributions were presented in Theorems 3 and 4, respectively.

### 7.2. The Load on the Tagged BS

In addition to the load on the typical BS, the load on the tagged BS is also important to understand (especially when we are interested in the performance of the typical user that is located in the tagged cell). Owing to the stationarity of Ψ, without loss of generality, we assume that the typical user is located at the origin. Due to the association law described above, this user will be associated with the zero-cell of the underlying PV tessellation. The associated BS is termed the *tagged* BS. Therefore, the load on the tagged BS is exactly the load (number of points of Ψ) $M_{p}$ on the zero-cell of the PV tessellation as studied in the paper. Hence, we denote this load by the RV $M_{p}$. Its two approximate distributions are given in Theorems 5 and 6, respectively.

### 7.3. Rate Coverage

Let us consider the typical vehicular user located at the origin. It is associated with the tagged BS located at distance *R*. Let all the BSs transmit at the same power, which is considered unity without loss of generality. Furthermore, we assume that a BS with zero load remains silent and hence does not cause interference at the typical user. We assume the standard path-loss model [1]. For this setting, the signal-to-interference ratio (SIR) of the typical user is

$$\begin{array}{c} \mathrm{SIR}=\frac{h_{0}R^{-\alpha }}{\sum_{y\in \Phi^{{}^{\prime }}}h_{y}{∥y∥}^{-\alpha }}, \end{array}$$

where $\Phi^{{}^{\prime }}$ is PP consisting of active BSs with density $\lambda_{a}$, α is the path loss exponent, $h_{0}$ and $h_{y}$, respectively, denote the fading gains of the link from the serving BS to the typical user and the BS at y to the typical user. Further, we assume Rayleigh fading. As a consequence, the fading coefficients *h* are exponentially distributed with unit mean. Assuming that the bandwidth *B* is equally shared by all users associated with the tagged BS, the achievable rate of the typical user is given by

(39) $$\begin{array}{c} R=\frac{B}{\left(M_{p}+1\right)}\log_{2}\left(1+\mathrm{SIR}\right). \end{array}$$

A key performance indicator for such networks is the *rate coverage* of the typical user, which is defined as the probability that the achievable rate by the typical user is greater than a certain threshold τ. Hence, the rate coverage for the typical receiver is defined as

$$\begin{array}{c} R_{c}\left(\tau \right)=P\left(R>\tau \right). \end{array}$$

Now, the probability that a BS is *active* is $p_{\mathrm{on}}=1-P\left[S_{p}=0\right]$. Using this, we can approximate the PP of active BSs as a PPP with density $\lambda p_{\mathrm{on}}$ [1,29]. Hence, the SIR coverage can be derived as [29]

(40) $$\begin{array}{cc} \; & p_{c}\left(\tau \right)=P\left[\mathrm{SIR}>\tau \right] \end{array}$$

(41) $$\begin{array}{cc} \; & =2\pi \lambda \int_{0}^{\infty }r\exp\left(-\lambda \pi r^{2}-p_{\mathrm{on}}\int_{r}^{\infty }\frac{2\pi \lambda \tau ydy}{\tau +{\left(y/r\right)}^{\alpha }}\right)dr\overset{\left(a\right)}{=}\frac{1}{1+p_{\mathrm{on}}\int_{1}^{\infty }\frac{dt}{1+t^{\alpha /2}\tau^{-1}}}, \end{array}$$

where (*a*) is achieved by substituting $\lambda \pi y^{2}=u$ and u=vt. Now using (39)–(41), we obtain the following result.

**Theorem** **7.** *The rate coverage for the typical user is*

(42) $$\begin{array}{cc} \; & R_{c}\left(\theta \left(\tau \right)\right)=\sum_{m=0}^{\infty }P\left(M_{p}=m\right)\frac{1}{1+p_{\mathrm{on}}\int_{1}^{\infty }\frac{dt}{1+t^{\alpha /2}\theta {\left(\tau \right)}^{-1}}}, \end{array}$$

*where $\theta \left(\tau \right)=2^{\frac{(m+1)\tau }{B}}-1$.*

We can now approximate $P(M_{p}=m)$ by $P({\hat{M}}_{p}=m)$ or $P({\tilde{M}}_{p}=m)$ to immediately obtain reasonable approximations for the above result.

#### 7.3.1. Meta Distribution of Rate Coverage

The rate coverage, as defined above, averages over all sources of randomness simultaneously regardless of the scale at which they are changing. While this results in a convenient metric, it is often desirable to obtain more fine-grained information about the variability of performance across the network. A rigorous way of doing that is through meta distributions [30]. Interested readers are advised to refer to [31,32] for a pedagogical treatment of this concept. For the purpose of this paper, it is sufficient to understand that the meta distribution of the rate coverage is defined as the probability that the typical vehicle’s conditional rate coverage (conditional on the PP realization) at a certain threshold τ is greater than a certain reliability target *x*, i.e., [30,31,32]

$$\begin{array}{c} {\bar{F}}_{P_{r}\left(\tau \right)}\left(x\right)=P\left(P_{r}\left(\tau \right)>x\right),\;\forall x\in \left[0,\;1\right], \end{array}$$

where $P_{r}\left(\tau \right)$ is the probability that achievable rate R at the typical user is greater than threshold τ conditioned on a realization of the BS PP Φ and load $M_{p}$ on the tagged BS, i.e.,

$$\begin{array}{c} P_{r}\left(\tau \right)=P\left(R>\tau |M_{p},\Phi \right). \end{array}$$

It is difficult to directly derive the meta-distribution of the rate coverage. Hence, first, we derive the moments of $P_{r}\left(\tau \right)$, and then, using the Gill Pelaez inversion theorem [33], we derive the meta distribution $\bar{F}\left(t,\tau \right)$ of rate coverage. The *q*th moment of $P_{r}\left(\tau \right)$ is related to the meta distribution of rate coverage $\bar{F}\left(t,\theta \right)$ as

(43) $$\begin{array}{cc} \; & M_{q}\left(\tau \right)=E\left[{\left(P_{r}\left(\tau \right)\right)}^{q}\right]=\int_{0}^{1}qt^{q-1}\bar{F}\left(t,\tau \right)dt. \end{array}$$

Note, that for q=1, $M_{1}\left(\tau \right)$ denotes the coverage probability.

**Theorem** **8.** *The q-th moment of the downlink coverage probability and the qth moment of rate coverage, respectively, is*

(44) $$\begin{array}{cc} \; & M_{q}\left(\tau \right)=\int_{u=0}^{\infty }\exp\left(-2p_{\mathrm{on}}u\int_{z=0}^{1}\left(1-\frac{1}{{\left(1+\tau z^{\alpha }\right)}^{q}}\right)\frac{dz}{z^{3}}\right)e^{-u}du \end{array}$$

(45) $$\begin{array}{cc} \; & S_{q}\left(\theta \left(\tau \right)\right)=\sum_{m=0}^{\infty }P\left(M_{p}=m\right)M_{q}\left(\theta (\tau )\right), \end{array}$$

*where $\theta \left(\tau \right)=2^{\frac{(m+1)\tau }{B}}-1$.*

The proof of the above theorem is provided in Appendix J.

Using Theorem 8, the definition of meta distribution and Gil-Pelaez lemma inversion theorem [33], we can derive the meta distribution for the rate coverage which is given in the following theorem. The proof is given in Appendix K.

**Theorem** **9.** *The meta distribution for rate coverage is*

(46) $$\begin{array}{cc} {\bar{F}}_{P_{r}\left(\tau \right)}\left(x\right) & =\frac{1}{2}-\frac{1}{\pi }\sum_{m=0}^{\infty }P\left(M_{p}=m\right)\int_{t=0}^{\infty }\frac{\sin\left(t\ln(x)+\Theta (t,\theta (\tau ))\right)}{\sqrt{{\left(f_{1,r}\left(t,\theta \left(\tau \right)\right)+1\right)}^{2}+{\left(f_{1,i}\left(t,\theta \left(\tau \right)\right)\right)}^{2}}}\frac{dt}{t}, \end{array}$$

*where*

$$\Theta \left(t,\theta \left(\tau \right)\right)=\tan^{-1}\left(\frac{f_{1,i}\left(t,\theta \left(\tau \right)\right)}{f_{1,r}\left(t,\theta \left(\tau \right)\right)+1}\right),$$

*and $f_{r}\left(t,\theta \left(\tau \right),z\right)=\cos\left(t\ln\left(1+\theta \left(\tau \right)z^{\alpha }\right)\right),\;f_{i}\left(t,\theta \left(\tau \right),z\right)=\sin\left(t\ln\left(1+\theta \left(\tau \right)z^{\alpha }\right)\right)$ and $f_{1,i}\left(t,\theta \left(\tau \right)\right)=2p_{\mathrm{on}}\int_{z=0}^{1}f_{i}\left(t,\theta \left(\tau \right),z\right)\frac{dz}{z^{3}}$ and $f_{1,r}\left(t,\theta \left(\tau \right)\right)=2p_{\mathrm{on}}\int_{z=0}^{1}\left(1-f_{r}\left(t,\theta \left(\tau \right),z\right)\right)\frac{dz}{z^{3}}$.*

#### 7.3.2. β Approximation for the Meta Distribution

Another simple yet tractable approach is to use the β approximation to obtain the meta distribution [30]. For this, we can use $S_{q}\left(\theta \left(\tau \right)\right)$ for q=1,2 to obtain the meta distribution as [30,34] ${\bar{F}}_{P_{r}\left(\tau \right)}\left(x\right)$

$$\begin{array}{cc} \; & =1-I_{x}\left(\frac{S_{1}\left(\theta \left(\tau \right)\right)\left(S_{1}\left(\left(\theta \left(\tau \right)\right)\right)-S_{2}\left(\theta \left(\tau \right)\right)\right)}{S_{2}\left(\theta \left(\tau \right)\right)-S_{1}^{2}\left(\theta \left(\tau \right)\right)},\frac{\left(S_{1}\left(\theta \left(\tau \right)\right)-S_{2}\left(\theta \left(\tau \right)\right)\right)\left(1-S_{1}\left(\theta \left(\tau \right)\right)\right)}{S_{2}\left(\theta \left(\tau \right)\right)-S_{1}^{2}\left(\theta \left(\tau \right)\right)}\right), \end{array}$$

where $I_{x}\left(\;\cdot \;\right)$ is the regularized incomplete beta function and $S_{1}$ and $S_{2}$ are given as

$$\begin{array}{cc} \; & S_{1}\left(\theta \left(\tau \right)\right)=\sum_{m=0}^{\infty }P\left({\tilde{M}}_{p}=m\right)M_{1}\left(\theta \left(\tau \right)\right)\;,S_{2}\left(\theta \left(\tau \right)\right)=\sum_{m=0}^{\infty }P\left({\tilde{M}}_{p}=m\right)M_{2}\left(\theta \left(\tau \right)\right). \end{array}$$

Now, we present the two important applications where the distance distribution can be directly used for the analysis of vehicular networks modeled by PLP-PPP.

### 7.4. Coverage under V2I Line of Sight (LOS) Only Communication

**Figure 5:**
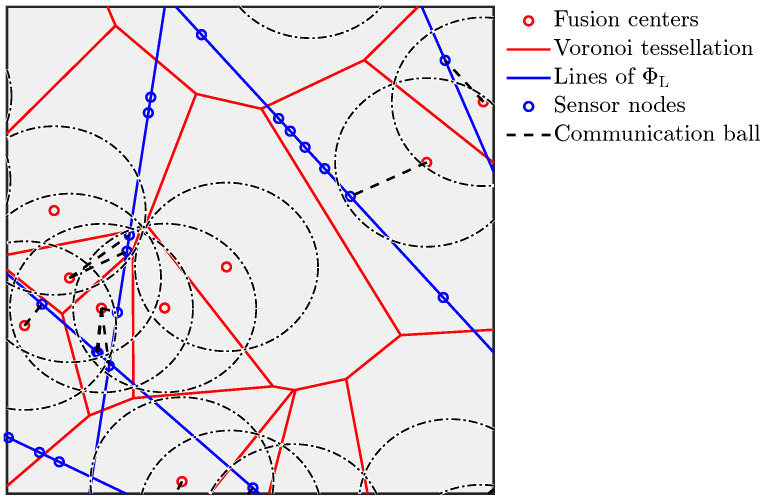
Illustration showing a graph connecting sensor nodes to associated fusion nodes under minimum distance based association and finite communication range.

In a vehicular network, BS may transmit messages to the other vehicles to communicate various data including critical updates and traffic information [35]. Various blockages can block a link making it non-LOS (NLOS). The wireless propagation along urban roads is usually different for LOS and NLOS links [36] (Figure 5). Especially at higher frequencies, NLOS may result in total loss of connection. The probability that a link with length *r* is LOS is given as $p_{L}\left(r\right)=e^{-\gamma r}$ where γ is the blockage parameter [1] dependent on blockage density. Now, consider V2I communication between a BS and its *k*-th nearest vehicle. If this link is LOS, the corresponding signal to noise ratio (SNR) is

(47) $$\begin{array}{c} {\mathrm{SNR}}_{k}=\frac{R_{k}^{-\alpha }}{N_{o}}. \end{array}$$

Here, $R_{k}$ is the distance of the *k*-th nearest vehicle from the BS and hence, has the distribution equal to that of the *k*-th CD of a PLP-PPP. The probability that this link is LOS and it is in coverage (i.e., SNR is above the threshold τ) is given as

(48) $$\begin{array}{cc} p_{c}\left(\tau \right) & =P\left[{\mathrm{SNR}}_{k}\geq \tau ,\mathrm{link}\;\mathrm{is}\;\mathrm{LOS}\right]=E\left[1\left(\frac{R_{k}^{-\alpha }}{N_{o}}\geq \tau \right)\exp\left(-\gamma R_{k}\right)\right]\\ \; & =\int_{r=0}^{{\left(\tau N_{o}\right)}^{-\frac{1}{\alpha }}}\exp\left(-\gamma r\right)f_{R_{k}}\left(r\right)dr, \end{array}$$

which can be computed using the CD distribution derived earlier in (18).

### 7.5. Content Caching in V2V within Communication Range $R_{c}$

As the number of vehicles on the road increases, the data content requirements for various vehicular applications and services increase commensurately. According to [37,38], the majority of mobile multimedia traffic consists of duplicate downloads of a limited number of popular content files. As current automobiles have large storage capacities, it is possible to cache frequently accessed files closer to the user [39]. Furthermore, in self-driving cars, the information related to traffic may be learned through the nearby vehicles using decentralized learning [40]. Therefore, to reduce the file access time and data dependency on the BS the vehicles may implement data caching, where a vehicle can access the data available at other vehicles within its communication range $R_{c}$ [27]. To characterize the performance of such networks, we can define a metric termed *cache hitting probability* as the probability that at least *k* neighbors are in the communication range of the typical vehicle. This gives

(49) $$\begin{array}{c} f_{k}=P^{o!}\left[R_{k}^{{}^{\prime }}\leq R_{c}\right]=F_{R_{k}^{{}^{\prime }}}\left(R_{c}\right), \end{array}$$

where $R_{k}^{\prime }$ is the *k*-th NND and $P^{o!}\left[\;\cdot \;\right]$ denotes the probability under the reduced Palm distribution. Further, we can simplify $f_{k}$ using the Corollary 5.

## 8. Application Area 2: Wireless Sensor Networks

As the second representative application area, we consider a wireless sensor network (WSN), which is a popular choice for sensing vast areas where human interventions are mostly limited or unwanted, such as forests. Let us consider a WSN where the sensors are deployed over a set of lines (for example in a forest along the trails) forming a PLP-PPP. Let PPP Φ denote the locations of the fusion centers that compile information coming from these sensors for data collection, monitoring, organizing, or taking further action. For example, in the barrier coverage [41] application, where the sensors are deployed by dropping along a line using an aircraft, the sensors’ data may be collected using a network of unmanned aerial vehicles (UAVs) distributed as a 2D PPP. To minimize power consumption, it is reasonable to assume that the sensors connect to their nearest fusion nodes. This results in a graph connecting sensor nodes to associated fusion nodes which is equivalent to G discussed in the paper. Hence, the number of sensors each fusion center needs to control can be seen as the load on the fusion center which is given as the number of sensors falling in its serving region. The information about this load will be useful for proper dimensioning of the system, such as determining the system bandwidth as well as the power requirement at the fusion centers. We can see that the distribution of this load is the same as the approximate load distribution given in Theorems 3 and 4.

Now, let us inspect the uplink connection carrying data from the sensors to the connected fusion node. Owing to limited capability and energy constraints, we can realistically assume that each sensor node can connect with a fusion center within a certain range around it. The radius $R_{s}$ of this communication disk (ball) depends on the sensors’ transmit power and the path loss. Hence, the *i*-th fusion node’s serving region $Y_{i}$ is given by the intersection of its Voronoi cell $C_{y_{i}}$ with its communication ball $b_{2}\left(y_{i},R_{s}\right)$. This results in a slightly different graph connecting sensor nodes to associated fusion nodes as shown in Figure 5. We can model the resultant tessellation using a germ grain process with fusion centers as germs and the corresponding serving regions as grains. Note that under the App2−typ approximation, the Voronoi cell is approximated as a ball $b_{2}\left(y_{i},R_{t}\right)$. Hence, the serving region is given as $Y_{i}=b_{2}\left(y_{i},R_{s}\right)\cap b_{2}\left(y_{i},R_{t}\right)=b_{2}\left(y_{i},\min\left(R_{s},R_{t}\right)\right)$ Hence, similar to Theorem 4, the distribution of the uplink load $S_{u}$ on the typical fusion center can be obtained as

(50) $$\begin{array}{c} P\left(S_{u}=m\right)=\int_{r_{t}=0}^{R_{s}}P\left(S(r_{t})=m\right)f_{R_{t}}\left(r_{t}\right)dr_{t}. \end{array}$$

Due to the limited communication range, some of the sensors may not be able to send their data to fusion centers. It is evident that a sensor is able to communicate if it falls inside the serving region of a fusion center which is equivalent to the event that it falls in the communication ball of at least one fusion center. For the setup described above, this probability is simply $1-\exp(-\pi \lambda R_{s}^{2})$.

Clearly, one can map the above setup consisting of sensors and fusion centers with the model discussed in this paper. Hence all the foundational results are either directly applicable to this setting or can be applied with simple variations.

## 9. Numerical Results

In this section, we first validate the presented approximations of load distribution by comparing them with the exact results obtained from Monte Carlo simulations. We have used the numerical software MATLAB (R2023a) to obtain the numerical results. We kept the simulation window size at $\frac{400}{\lambda }$. For the load distribution results, we perform $2\times 10^{5}$ iterations with each iteration generating a PPP representing the BS deployment, selecting the typical and tagged cell, generating a PLP-PPP for user distribution and counting the number of points of PLP-PPP for load inside each of the two cells. Similarly, $2\times 10^{5}$ realizations of PLP-PPP are generated for getting the CDFs of *k*th CD and NND for PLP-PPP. Other parameter values, such as bandwidth, line density ($\lambda_{L}$), and BS density (λ), are declared wherever they are used. We present some useful results related to the network performance of the applications discussed above.

### 9.1. CD and NND Distributions

**Figure 6:**
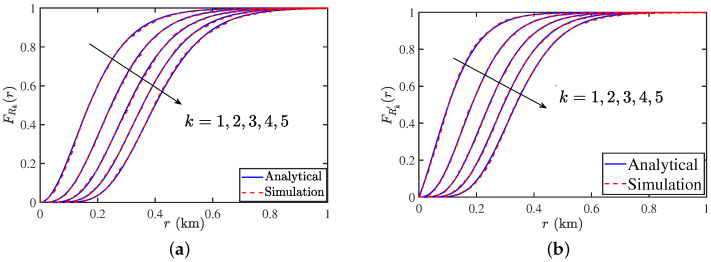
(**a**) CDF of *k*-th CD for PLP-PPP. (**b**) CDF of *k*-th NND for the PLP-PPP. The parameters are $\lambda_{R}=2$ vehicle/km, $\lambda_{L}=5/\pi$/km and λ=1 $\mathrm{BS}/{\mathrm{km}}^{2}$.

In Figure 6a,b, we present the CDF of *k*-th CD and NND derived in this paper along with the results obtained from simulations. The purpose of this comparison is to simply verify the analysis for a variety of settings.

### 9.2. Validation of the Approximations Proposed in the Load Distributions

**Figure 7:**
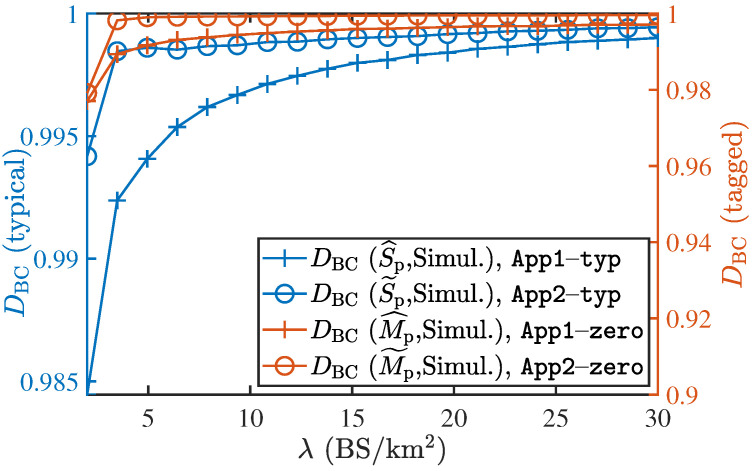
The analytical expressions obtained for the load distributions in both the typical and the zero-cell are accurate since the BC is close to 1.

To verify the proposed approximations, the BC of the approximate expression against the exact values (obtained via simulation) are presented in Figure 7. It can be readily observed that the BC for both the approximations is closer to 1 for both the typical and zero-cell cases. Additionally, we see that App2−typ and App2−zero are closer to 1 than App1−typ and App1−zero, respectively. Since App2−typ and App2−zero approximations are also simpler and easier to deal with, we will use them in the subsequent numerical analyses. Furthermore, we observe that BC increases with an increase in the BS density which indicates that the accuracy of the approximations improves as the BS density increases.

### 9.3. Mean and Variance of the Load on the Typical and Zero-Cell

**Figure 8:**
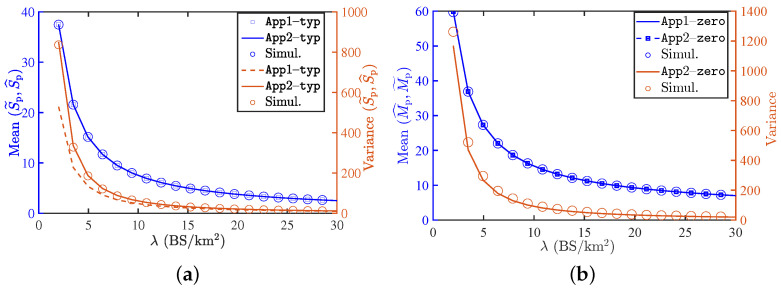
(**a**) Plot of the mean and variance of the load on the typical cell. (**b**) Plot of the mean and variance of the load on the zero-cell. It can be observed that App2−typ is more accurate compared to App1−typ. The parameters are $\lambda_{L}=5/\pi$ ${\mathrm{km}}^{-1}$, $\lambda_{R}=15$ vehicle/km.

The mean and variance of the load in the typical cell under both approximations are plotted in Figure 8a, along with their respective exact values (i.e., obtained via simulations). Both the results are remarkably accurate for both the typical and the zero-cells. The mean load obtained under both approximations is accurate. The variance values obtained under App2−typ are accurate for all values of λ, whereas the values obtained under App1−typ exhibit slight differences from the respective simulation-based values in the regime of smaller λ.

Similarly, the mean and variance of the zero-cell load under approximations are shown in Figure 8b. As was the case above, the variance under App2−zero is accurate for all values of λ. We have omitted the variance result for App1−zero since it is not as accurate as the result obtained under App2−zero.

### 9.4. Validation of Rate Coverage and Meta Distribution

**Figure 9:**
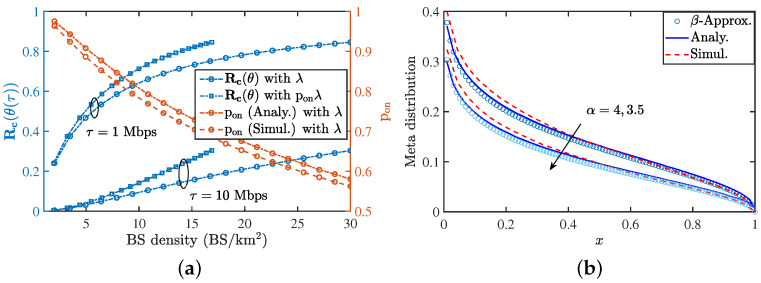
(**a**) Plot showing the rate coverage for two different values of threshold, it also shows the probability that a BS is active with varying BS density. We consider α=3.5, B=20 MHz. (**b**) presents the meta distribution for the PLP-PPP process with $p_{\mathrm{on}}=1$. The parameters are $\lambda_{L}=5/\pi$ ${\mathrm{km}}^{-1}$, $\lambda_{R}=15$ vehicles/km, $\lambda =2\mathrm{BS}/{\mathrm{km}}^{2}$ and τ=1 MHz. For both the plots, the load distribution on the zero-cell is obtained under App2−zero.

Figure 9a shows the variation of the rate coverage with respect to the BS density for two different values of threshold. We have also plotted the *active probability* with the BS density λ. We see that the active probability obtained using App2−typ closely approximates the true results obtained from simulations. With an increase in the BS’s density, the active probability reduces and hence, more BSs remain silent, which reduces the power consumption. Figure 9b presents the meta distribution for the rate coverage. We have also shown the values obtained via the beta approximation.

### 9.5. Comparison with PPP Based Models

**Figure 10:**
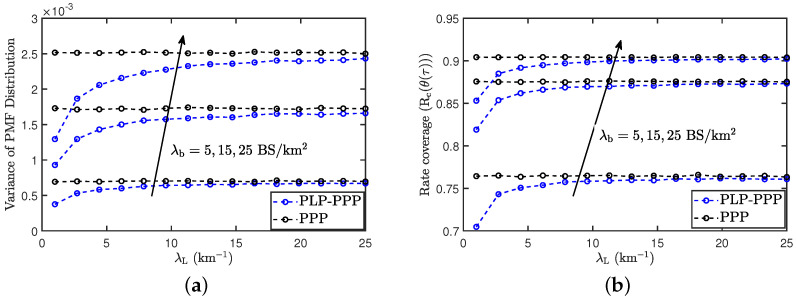
(**a**) Variance of the load distribution vs the line density for different values of the BS density for the cases where users are modeled using PLP-PPP and PPP (**b**) the rate coverage vs line density for different values of the BS density for the cases where users are modeled using PLP-PPP and PPP. The vehicular density μ=25 vehicle/km, bandwidth B=20 MHz, α=4 and τ=1 MHz.

Please note that PLP-PPP can be approximated using a 2D PPP to reduce complexity at the cost of accuracy. It was shown in [15] (Theorem-1) that the PLP-PPP converges to a PPP with the same density μ asymptotically when the line density of PLP-PPP $\lambda_{L}\to \infty$ while keeping the density μ constant. Hence, such approximation is accurate for a high line density $\lambda_{L}$. However, such an approximation may not be valid at lower line density. To further investigate it, in Figure 10a, we show the variance of load distribution in a vehicular communication network, where vehicular users are distributed as PLP-PPP and PPP, respectively, with respect to the road density $\lambda_{L}$ while keeping total vehicular density constant and the same for the two cases. For higher line density, vehicles in a single cell are still spread over many roads and hence PLP-PPP resembles PPP. Hence, we observe that both PPP and PLP-PPP provide the same load distribution. However, for lower line density, usually only a single line falls inside a cell. Hence, the variance reduces for PLP-PPP. Further, in Figure 10b, we compare the rate coverage of PLP-PPP and PPP distributed vehicular users with respect to the road density $\lambda_{L}$ while keeping total vehicular density the same. We observe that for higher line density, both PPP and PLP-PPP provide the same rate coverage. However, for lower line density, rate coverage decreases. This discussion highlights the importance of PLP-PPP-based models. As stated earlier, PLP-PPP can be approximated by PPPs to simplify analysis; however, such models may have lower accuracy, especially at lower line densities.

### 9.6. SNR Distribution and Cache Hitting Probability

**Figure 11:**
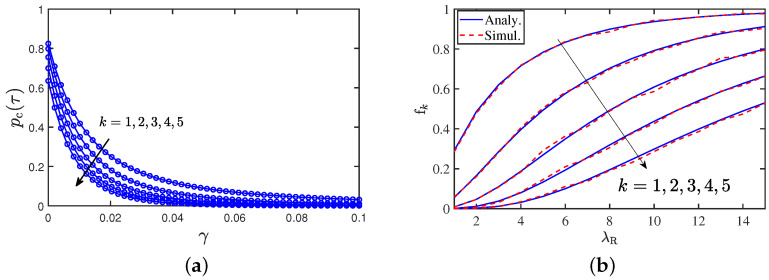
(**a**) Probability that the LOS link is in coverage as a function of blockage probability with $N_{o}=10^{-8}$ W, α=3.5, τ=1 dB, $\lambda_{R}=15$ vehicles/km and the road density $\lambda_{L}=5/\pi$ ${\mathrm{km}}^{-1}$. (**b**) Variation of the cache hitting probability with road vehicular density $\lambda_{R}$. The road density $\lambda_{L}=5/\pi$ ${\mathrm{km}}^{-1}$, the broadcast range $R_{c}=100$ m and λ=1 $\mathrm{BS}/{\mathrm{km}}^{2}$.

The SNR distributions for the ten closest LOS vehicles from the typical BS are shown in Figure 11a. We observe a significant difference between the SNR distributions for the closest and second-closest vehicles, but the difference between SNR distributions from *k* and k+1-th closest vehicles decreases with the order *k*. In Figure 11b, the cache content probability is shown with respect to the vehicular density $\lambda_{R}$. We observe that in a situation with heavy traffic (growing vehicle density), the cache striking probability increases. With an increase in $f_{k}$, the content is made accessible to nearby vehicles, which may lower the burden on the BSs in a scenario with heavy traffic. Thus, content caching and broadcasting could be potentially advantageous in high-traffic situations.

## 10. Conclusions

In this paper, we explored several important properties of the PLP-PPP as well as its *random bipartite geometric associator graph* in which each point of the PLP-PPP connects with its closest point of an independent PPP. This graph is equivalent to partitioning the PLP-PPP with a PVT formed by an independent PPP. Key contributions related to PLP-PPP involve the distributions of its *k*-th CD and NND. We then presented an empirical distribution for the perimeter distribution of the zero-cell of the aforementioned PVT. The accuracy of this result is validated by using the BC coefficient for a range of values of the PPP density. Additionally, we presented the distribution of the length of any randomly selected chord of a zero-cell of this PVT. Using these results, we provided two approximate distributions of the node degree of the associator graph mentioned above. We then applied these results to several wireless network settings. For instance, we presented the distributions of the load on the typical and the tagged BS in a vehicular network in which the vehicles are modeled as a PLP-PPP and the BSs are modeled as an independent PPP (and each vehicle connects to its closest BS). Using this load distribution, we then presented the rate coverage and the meta-distribution of the rate coverage for a vehicle. We also presented the SNR coverage probability for a vehicle and content hitting probability using the *k*-th CD and NND of PLP-PPP, respectively. We numerically compared the PLP-PPP and PPP-based models to discuss the accuracy vs complexity trade-off. We concluded the paper by rigorously demonstrating that the results of this paper can also be applied to wireless sensor networks in which sensors are deployed over a set of lines (for example in a forest along the trails) forming a PLP-PPP.

## Figures and Tables

**Figure A1:**
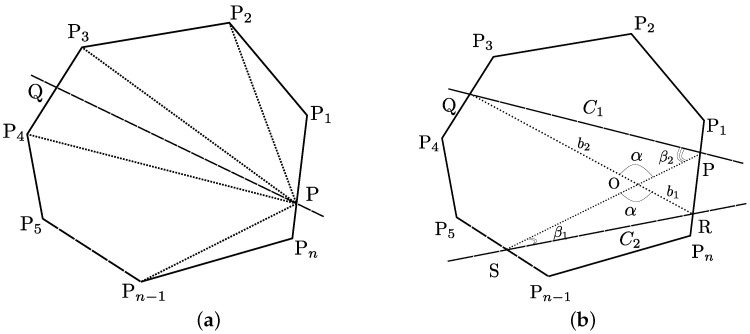
(**a**) An irregular convex polygon with perimeter *Z* and an arbitrary chord PQ. (**b**) An irregular convex polygon with perimeter *Z* featuring two non intersecting chords PQ and RS.

## Data Availability

No new data were created or analyzed in this study. Data sharing is not applicable to this article.

## Appendix A. Proof of Proposition 1

We consider an irregular convex polygon with perimeter *Z* as shown in the Figure A1a. Let there be an arbitrary chord intersecting the polygon at two points P and Q. Now using the triangular inequality, we can obtain the following inequalities

(A1) $$\begin{array}{c} |PP_{1}\left|+\right|P_{1}P_{2}|\;\geq \;|PP_{2}|,\;|PP_{2}\left|+\right|P_{2}P_{3}|\;\geq \;|PP_{3}|,\;|PP_{3}\left|+\right|P_{3}Q|\;\geq \;|PQ|. \end{array}$$

From the above, it can be concluded that

(A2) $$\begin{array}{cc} \; & |PP_{1}\left|+\right|P_{1}P_{2}|+P_{2}P_{3}+P_{3}Q\;\geq \;\left|PQ\right|. \end{array}$$

Similarly, we can prove that

(A3) $$\begin{array}{cc} \; & |QP_{4}\left|+\right|P_{4}P_{5}\left|+\ldots +\right|P_{n-1}P_{n}\left|+\right|P_{n}P|\;\geq \;|PQ|. \end{array}$$

Taking the summation of (A2) and (A3), we obtain the following

(A4) $$\begin{array}{cc} \; & 2|PQ|\;\leq \;|P_{1}P_{2}\left|+\right|P_{2}P_{3}\left|+\ldots +\right|P_{n-1}P_{n}|,\Rightarrow 2|PQ|\leq Z, \end{array}$$

which completes the proof of Proposition 1.

## Appendix B. Proof of Proposition 2

Consider the polygon shown in Figure A1b. Now consider two non intersecting chords PQ and RS of lengths $C_{1}$ and $C_{2}$, respectively. To prove this result, we will show that the length of the chord RS depends on $C_{1}$. Let us join the opposite edges of chords to create line segments PS and RQ. By applying the sine rule to the triangle OPQ, we get

$$\begin{array}{c} \frac{C_{1}}{\sin\alpha }=\frac{b_{2}}{\sin\beta_{2}}\Rightarrow \sin\alpha =\frac{C_{1}\sin\beta_{2}}{b_{2}}. \end{array}$$

Similarly, by applying the sine rule to the triangle OSR, we get

$$\begin{array}{cc} \; & \frac{C_{2}}{\sin\alpha }=\frac{b_{1}}{\sin\beta_{1}}\Rightarrow C_{2}=\frac{b_{1}}{\sin\beta_{1}}\frac{C_{1}\sin\beta_{2}}{b_{2}}\Rightarrow C_{2}=C_{1}\frac{R_{1}}{R_{2}}, \end{array}$$

where $R_{1}$ and $R_{2}$ are the radii of the circumcircles of triangles OPQ and ORS, respectively. Furthermore, these circumcircles fall inside the convex polygon, and therefore, the perimeters of both the circumcircles are upper bounded by the perimeter of polygon [42], which is assumed to be *Z*. Therefore, the ratio $R_{1}/R_{2}$ is dependent on *Z*. Consequently, $C_{2}$ is proportional to $C_{1}$, which establishes that $C_{1}$ and $C_{2}$ are dependent RVs.

## Appendix C. Proof of Corollary 2: Proof of Length Distribution of Tagged Chord

To derive the PDF $f_{\tilde{C}}\left(\tilde{c}\right)$, we use the fact that the area of a zero-cell is larger than the area of the typical cell. Hence, $f_{\tilde{C}}\left(\tilde{c}\right)$ is a scaled version of $f_{C}\left(c\right)$ and can be written as

(A5) $$\begin{array}{c} f_{\tilde{C}}\left(\tilde{c}\right)\approx \eta f_{C}\left(\eta c\right). \end{array}$$

To obtain the approximate value of η, we equate the mean load on the tagged BS obtained by two methods. The first method is using the stationarity property of PP [1]. The second method is using the PGF of ${\hat{M}}_{p}$. In the first method, we know that the average number of vehicles falling in the zero-cell is the product of the density μ of Ψ and the average size of the zero-cell [43]. Hence, the mean is equal to $\frac{1.28\mu }{\lambda }$. In the second method, we observe from Corollary 8 that the mean of ${\hat{M}}_{p}$ is the summation of the mean number of points falling in the zero-cell which is equal to $\frac{\mu }{\pi }E\left[\tilde{C}\right]E\left[Z^{\prime }\right]$ and the mean number of points on the tagged chord. Therefore, equating $\frac{\mu }{\pi }E\left[\tilde{C}\right]E\left[Z^{\prime }\right]$ with $\frac{1.28\mu }{\lambda }$, we get

$$\begin{array}{cc} \; & \frac{\mu }{\pi }E\left[\tilde{C}\right]E\left[Z^{\prime }\right]=1.28\frac{\mu }{\lambda }\overset{\left(a\right)}{\Rightarrow }E\left[\tilde{C}\right]\frac{4.4906}{\sqrt{\lambda }}=\frac{1.28\pi }{\lambda }\Rightarrow E\left[\tilde{C}\right]=\frac{1.28\pi }{4.4906\sqrt{\lambda }},\\ \; & \frac{1}{\eta }\frac{\pi }{4\sqrt{\lambda }}=\frac{1.28\pi }{4.4906\sqrt{\lambda }}, \end{array}$$

where (a) is obtained by replacing the mean of $Z^{{}^{\prime }}$ obtained in (10). Solving further, we obtain the value of η.

## Appendix D. Proof of Theorem 1: PGF and Corresponding PMF of *S*(*r*)

To obtain the PGF of the number of points of Ψ falling in an arbitrary ball of radius *r*, we first compute the PGF of the number of points on each line of PLP that intersects the ball. Due to independence among the lines of PLP, we take the product of the PGFs conditioned on the number of lines *N* that are intersecting the ball. Since *N* is a Poisson RV, we derive the PGF by deconditioning with the distribution of *N*. Hence the number of points S(r) is

$$\begin{array}{c} S\left(r\right)=\sum_{l_{k}\in \Phi_{L}}\Psi_{l_{k}}\left(b_{2}\left(o,r\right)\right). \end{array}$$

The length of the segment of road l(ρ,ϕ) is $2\sqrt{r^{2}-\rho^{2}}$. Hence, the number of vehicles on l(ρ,ϕ) have Poisson distribution with mean $2\lambda_{R}\sqrt{r^{2}-\rho^{2}}$. Therefore, conditioned on ρ the PGF for number of points falling inside $b_{2}\left(o,r\right)$ is

$$P_{\Psi_{l_{k}}\left(b_{2}\left(o,r\right)\right)|\rho }\left(s,\sqrt{r^{2}-\rho^{2}}\right)=e^{2\lambda_{R}\sqrt{r^{2}-\rho^{2}}\left(s-1\right)}.$$

Note that, ρ is a uniform RV in the range [−r,r]. Hence, deconditioning over ρ the PGF expression reduces to

$$\begin{array}{c} P_{S(r)}\left(s,r\right)=\frac{1}{2r}\int_{-r}^{r}e^{\left(2\lambda_{R}\sqrt{r^{2}-\rho^{2}}\left(s-1\right)\right)}d\rho \overset{\left(a\right)}{=}\frac{1}{r}\int_{0}^{r}e^{\left(2\lambda_{R}\sqrt{r^{2}-\rho^{2}}\left(s-1\right)\right)}d\rho , \end{array}$$

where step (a) follows from a definite integral property. Let there be *N* such lines intersecting $b_{2}\left(o,r\right)$, hence the joint PGF is the product of individual PGFs which is equal to

$$\begin{array}{c} P_{S(r)|N=n}\left(s,r\right)={\left(\frac{1}{r}\int_{0}^{r}e^{\left(2\lambda_{R}\sqrt{r^{2}-\rho^{2}}\left(s-1\right)\right)}d\rho \right)}^{n}. \end{array}$$

Here, *N* is a Poisson RV with mean $\lambda_{L}2\pi r$. From the law of total probability, deconditioning over *N*, we obtain the PGF as

$$\begin{array}{cc} P_{S(r)}\left(s\right) & =\exp\left(2\pi \lambda_{L}\left(\int_{0}^{r}\exp\left(2\lambda_{R}\sqrt{r^{2}-\rho^{2}}\left(s-1\right)\right)d\rho -r\right)\right). \end{array}$$

## Appendix E. Proof of Theorem 2: The PGF and Corresponding PMF for *M*(*r*)

Without loss of generality, we assume that the typical point of Ψ is located at the origin. Hence the number of points of Ψ falling inside a circle of radius *r* conditioned on o∈Ψ can be divided into two parts. The first is the number of points falling on the line passing through the origin of length 2r, for which the PGF is $e^{2\lambda_{R}r\left(s-1\right)}$. The second is the number of points of Ψ falling in ball $b_{2}\left(o,r\right)$ for which the PGF is provided in Theorem 1. Taking the product of the two PGFs, we obtain the desired PGF.

## Appendix F. Proof of Theorem 3: Proof of PGF, PMF, Mean and Variance of ${\hat{S}}_{p}(\mathrm{App}1-\mathrm{type})$

The number of points falling in the typical Voronoi cell $V_{t}$ is

$${\hat{S}}_{p}=\sum_{l_{k}\in \Phi }\psi_{l_{k}}\left(V_{t}\right).$$

Let *N* be the number of chords intersecting the typical Voronoi cell. The PGF expression is

$$\begin{array}{cc} P_{{\hat{S}}_{p}|N=n}\left(s\right) & =E_{\Psi }\left[s^{{\hat{S}}_{p}}|N=n\right]=E_{\Psi }\left[\prod_{k=1}^{n}s^{\psi_{l_{k}}\left(V_{t}\right)}|N=n\right]={\left[\int_{0}^{\infty }e^{h(s,c)}f_{C}\left(c\right)dc\right]}^{n}, \end{array}$$

where $h\left(s,c\right)=\lambda_{R}c\left(s-1\right)$. Note that the number of chords *N* intersecting the typical Voronoi cell is a Poisson RV with mean $\lambda_{L}Z$, where *Z* denotes the perimeter of $V_{t}$. Hence, conditioned on Z=z, deconditioning with the distribution of *N*, we obtain the PGF as

(A6) $$\begin{array}{cc} \; & P_{{\hat{S}}_{p}|Z=z}\left(s\right)=\sum_{n=0}^{\infty }\frac{e^{-\lambda_{L}z}{\left(\lambda_{L}z\right)}^{n}}{n!}P_{{\hat{S}}_{p}|N}\left(s\right)=e^{-\lambda_{L}z\left(1-\int_{0}^{\infty }e^{h(s,c)}f_{C}\left(c\right)dc\right)}. \end{array}$$

Finally, deconditioning with the distribution of *Z* provided in (8), we obtain the PGF. Note that, the conditional PGF $P_{{\hat{S}}_{p}^{}|Z}\left(s\right)$ is in the form of $\exp\left(\;\cdot \;\right)$. Further to find the PMF, we need the *k*-th derivative of conditional PGF. After obtaining the *k*-th derivative, we obtain the PMF of ${\hat{S}}_{p}$ as

(A7) $$\begin{array}{cc} \; & P\left[{\hat{S}}_{p}^{}=k\right]={\left[P_{{\hat{S}}_{p}^{}}^{\left(k\right)}\left(s\right)/k!\right]}_{s=0}=\left(1/k!\right)\int_{z=0}^{\infty }P_{{\hat{S}}_{p}|Z}^{\left(k\right)}\left(s\right)f_{Z=z}\left(z\right)dz. \end{array}$$

To derive the mean of ${\hat{S}}_{p}$, we need the first derivative of the PGF which is

$$\begin{array}{cc} \; & {\left[P_{{\hat{S}}_{p}}^{\left(1\right)}\left(s\right)\right]}_{s=1}=\int_{z=0}^{\infty }\lambda_{L}z\int_{c=0}^{\infty }\lambda_{R}cf_{C}\left(c\right)dcf_{Z}\left(z\right)dz=\lambda_{L}\lambda_{R}\int_{c=0}^{\infty }cf_{C}\left(c\right)dc\int_{z=0}^{\infty }zf_{Z}\left(z\right)dz. \end{array}$$

Solving further, we obtain the mean of ${\hat{S}}_{p}$. To obtain the variance of ${\hat{S}}_{p}$, we need the second derivative of $P_{{\hat{S}}_{p}}\left(s\right)$, which is given as

$$\begin{array}{c} P_{{\hat{S}}_{p}}^{\left(2\right)}\left(s\right)={\left(\lambda_{L}\lambda_{R}E\left[C\right]\right)}^{2}E\left[Z^{2}\right]+\lambda_{L}\lambda_{R}^{2}E\left[Z\right]E\left[C^{2}\right]. \end{array}$$

Using (1), we obtain the variance of ${\hat{S}}_{p}$.

## Appendix G. Proof of Corollary 7: Proof of Mean and Variance of ${\tilde{S}}_{p}$

The mean number of points of Ψ falling in ball $b_{2}\left(o,r_{t}\right)$ is $\mu \pi E[R_{t}^{2}]$. Substituting $E[R_{t}^{2}]=1/\pi \lambda$, we obtain the mean of ${\tilde{S}}_{p}$. To derive the variance of ${\tilde{S}}_{p}$, we need the second derivative of the PGF that is given as

$$\begin{array}{cc} \; & {\left[P_{{\tilde{S}}_{p}}^{2}\left(s\right)\right]}_{s=1}=\int_{r_{t}=0}^{\infty }\left[{\left(2\pi \lambda_{L}\left(2\lambda_{R}\right)\int_{t=0}^{r_{t}}\frac{t^{2}dt}{\sqrt{r_{t}^{2}-t^{2}}}\right)}^{2}+2\pi \lambda_{L}{\left(2\lambda_{R}\right)}^{2}\int_{t=0}^{r_{t}}\frac{t^{3}dt}{\sqrt{r_{t}^{2}-t^{2}}}\right]f_{R_{t}}\left(r_{t}\right)dr_{t}\\ \; & =\int_{r_{t}=0}^{\infty }\left[{\left(4\pi \lambda_{L}\lambda_{R}\frac{\pi }{4}r_{t}^{2}\right)}^{2}+8\pi \lambda_{L}\lambda_{R}^{2}\frac{2}{3}r_{t}^{3}\right]f_{R_{t}}\left(r_{t}\right)dr_{t}\\ \; & ={\left(\pi \mu \right)}^{2}E\left[r_{t}^{4}\right]+\frac{16}{3}\lambda_{R}\mu E\left[r_{t}^{3}\right]. \end{array}$$

Using (1) and the second derivative, we derive the variance of ${\tilde{S}}_{p}$.

## Appendix H. Proof of Theorem 5: Proof of PGF of ${\hat{M}}_{p}$

The number of points falling in the zero-cell is the sum of two RVs. First is the number of points falling on the tagged chord and second is the number of points falling on any other chords of zero-cell conditioned on the perimeter of the zero-cell. We further assume that the two RVs are independent hence we write their PGFs separately. The PGF for the number of points on the tagged chord is

(A8) $$\begin{array}{c} P_{1}\left(s\right)=\int_{c_{o}=0}^{\infty }e^{\lambda_{R}c_{o}\left(s-1\right)}f_{C_{o}}\left(c_{o}\right)dc_{o}. \end{array}$$

The PGF for the number of points falling in the zero-cell of perimeter $Z^{\prime }$ can be derived similar to Theorem 3. The PGF is given by

(A9) $$\begin{array}{c} P_{2}\left(s\right)=\int_{z^{{}^{\prime }}=0}^{\infty }\exp\left(-\lambda_{L}z^{{}^{\prime }}\left(1-\int_{0}^{\infty }\exp\left(\lambda_{R}c\left(s-1\right)\right)f_{\tilde{C}}\left(\tilde{c}\right)d\tilde{c}\right)\right)f_{Z^{\prime }}\left(z^{\prime }\right)dz^{\prime }. \end{array}$$

Taking the product of (A8) and (A9), we obtain the PGF of ${\hat{M}}_{p}$.

## Appendix I. Proof of Theorem 6: Proof for PGF of ${\tilde{M}}_{p}$

The ${\tilde{M}}_{p}$ is the sum of two independent RV, the first is the number of points falling on chord of length $c_{o}$ and the second is number of vehicles falling inside ball of radius $r_{o}$. Hence,

(A10) $$\begin{array}{cc} \; & {\tilde{M}}_{p}=\psi_{l_{o}}\left(b_{1}\left(o,c_{o}/2\right)\right)+\Psi \left(b_{2}\left(o,r_{o}\right)\right), \end{array}$$

where $\psi_{l_{o}}\left(b_{1}\left(o,c_{o}/2\right)\right)$ denotes the number of points on zero chord of length $c_{o}$ with PGF $\exp\left(\lambda_{R}c_{o}\left(s-1\right)\right)$ and $\Psi (b_{2}\left(o,r_{o}\right))$ denotes the number of points of Ψ falling inside a ball of radius $r_{o}$, and $|b_{2}\left(o,r_{o}\right)\left|=\right|V_{t_{o}}|$. The PGF of $\Psi (b_{2}\left(o,r_{o}\right))$ can be determined using Theorem 4. The product of these two PGFs provides the PGF of ${\tilde{M}}_{p}$.

## Appendix J. Proof of Theorem 8: Proof for *q*th Moment of Rate Coverage

The coverage probability conditioned on the nearest BS distance *R* is

$$\begin{array}{cc} P_{r}\left(\tau \right) & =E\left[P\left(\mathrm{SIR}>\tau \right)|\Phi ,R=r\right]=E\left[P\left(\frac{h_{0}r^{-\alpha }}{I}>\tau |\Phi ,R=r\right)\right]\\ \; & =E\left[P\left(h_{0}>\tau Ir^{\alpha }\right)|\Phi ,R=r\right]=\left[E\left[\exp\left(-\tau Ir^{\alpha }\right)|\Phi ,R=r\right]\right]\overset{\left(a\right)}{=}\prod_{y\in \Phi }\left[\frac{1}{1+\tau r^{\alpha }{\left||y|\right|}^{-\alpha }}|R=r\right], \end{array}$$

where (a) is obtained using the Laplace transform of interference [1]. Hence, the *q*th moment of $P_{r}\left(\tau \right)$ is

$$\begin{array}{cc} \; & M_{q}\left(\tau \right)=E_{\Phi }\left[\prod_{y\in \Phi }\frac{1}{{\left(1+\tau r^{\alpha }{\left||y|\right|}^{-\alpha }\right)}^{q}}|R\right]. \end{array}$$

Using the PGFL of PPP and deconditioning over *R*, we obtain the *q*-th moment of the coverage probability as

$$\begin{array}{cc} \; & 2\pi \lambda \int_{r=0}^{\infty }\exp\left(-2\pi p_{\mathrm{on}}\lambda \int_{r}^{\infty }\left(1-\frac{1}{{\left(1+\tau r^{\alpha }y^{-\alpha }\right)}^{q}}\right)ydy\right)e^{-\lambda \pi r^{2}}rdr, \end{array}$$

which can be further simplified by substituting r/y=z and $\lambda \pi r^{2}=u$, which completes the proof of Theorem 8. Further the *q*-th moment of the rate coverage is

$$\begin{array}{cc} \; & S_{q}\left(\theta \left(\tau \right)\right)=E\left[{\left(P\left(\left(R>\tau \right)|\Phi ,{\tilde{M}}_{p}\right)\right)}^{q}\right]=E\left[{\left(P\left(\frac{B}{{\tilde{M}}_{p}+1}\log_{2}\left(1+\mathrm{SIR}\right)>\tau \right)|\Phi ,{\tilde{M}}_{p}\right)}^{q}\right]\\ \; & =E\left[{\left(P\left(\left(\mathrm{SIR}>2^{\frac{({\tilde{M}}_{p}+1)\tau }{B}}-1\right)|\Phi ,{\tilde{M}}_{p}\right)\right)}^{q}\right]=\left[P\left(M_{q}\left(\theta (\tau )\right)|{\tilde{M}}_{p}\right)\right], \end{array}$$

where $\theta \left(\tau \right)=2^{\frac{({\tilde{M}}_{p}+1)\tau }{B}}-1$. Deconditioning over ${\tilde{M}}_{p}$, we obtain the *q*th moment of rate coverage probability.

## Appendix K. Proof of Theorem 9: Proof for the Meta Distribution of Rate Coverage

From the definition of the meta distribution of rate coverage, we get

(A11) F¯Pr(τ)(x)=P(Pr(τ)>x)=(a)12+1π∫t=0∞Ime−itln(x)Sit(θ(τ))tdt=(b)12+1π∑m=0∞P(M˜p=m)∫t=0∞Ime−itln(x)Mitθ(τ)tdt,

where step (a) is obtained using the Gill-Pelaez lemma for the inversion of the it-th moment of $P_{r}\left(\tau \right)$ which turns out to be the function of the it-th moment $S_{i\;t}\left(\theta \left(\tau \right)\right)$, of the rate coverage and step (b) is obtained by substituting $S_{i\;t}\left(\theta \left(\tau \right)\right)$ from (45). After step (b), we can further simplify by extracting the imaginary part of $e^{-i\;t\ln(x)}M_{i\;t}\left(\theta (\tau )\right)$. First, we determine the imaginary part of $M_{it}\left(\theta \left(\tau \right)\right)$. The $M_{it}\left(\theta \left(\tau \right)\right)$ is

(A12) $$\begin{array}{cc} \; & M_{it}\left(\theta \left(\tau \right)\right)=\int_{u=0}^{\infty }\exp\left(-2p_{\mathrm{on}}u\int_{0}^{1}\left(1-\frac{1}{{\left(1+\theta \left(\tau \right)z^{\alpha }\right)}^{i\;t}}\right)\frac{dz}{z^{3}}\right)e^{-u}du. \end{array}$$

Now in (A12) the term ${\left(1+\theta \left(\tau \right)z^{\alpha }\right)}^{-i\;t}$ can be written as

$$\begin{array}{cc} {\left(1+\theta \left(\tau \right)z^{\alpha }\right)}^{-i\;t} & =e^{-i\;t\ln\left(1+\theta \left(\tau \right)z^{\alpha }\right)}=\cos\left(t\ln\left(1+\theta \left(\tau \right)z^{\alpha }\right)\right)-i\;\sin\left(t\ln\left(1+\theta \left(\tau \right)z^{\alpha }\right)\right)\\ \; & =f_{r}\left(t,\theta \left(\tau \right),z\right)-i\;f_{i}\left(t,\theta \left(\tau \right),z\right). \end{array}$$

Substituting the above in (A12) and simplifying further, we get

$$\begin{array}{cc} \; & M_{it}\left(\theta \left(\tau \right)\right)=\int_{u=0}^{\infty }\exp\left(-2p_{\mathrm{on}}u\int_{z=0}^{1}\left(1-f_{r}\left(t,\theta \left(\tau \right),z\right)+i\;f_{i}\left(t,\theta \left(\tau \right),z\right)\right)\frac{dz}{z^{3}}\right)e^{-u}d\;u\\ \; & =\int_{u=0}^{\infty }\exp\left(-u2p_{\mathrm{on}}\int_{z=0}^{1}\left(1-f_{r}\left(t,\theta \left(\tau \right),z\right)\right)\frac{dz}{z^{3}}\right)\exp\left(-i\;u2p_{\mathrm{on}}\int_{z=0}^{1}f_{i}\left(t,\theta \left(\tau \right),z\right)\frac{dz}{z^{3}}\right)e^{-u}du. \end{array}$$

In order to provide a compact form of the equations, let $f_{1,i}\left(t,\theta \left(\tau \right)\right)=2p_{\mathrm{on}}\int_{z=0}^{1}\left(f_{i}\left(t,\theta \left(\tau \right),z\right)\right)\frac{dz}{z^{3}}$ and $f_{1,r}\left(t,\theta \left(\tau \right)\right)=2p_{\mathrm{on}}\int_{z=0}^{1}\left(1-f_{r}\left(t,\theta \left(\tau \right),z\right)\right)\frac{dz}{z^{3}}$. Now, we can easily extract the imaginary term, Ime−itln(x)Mitθ(τ), that appears in (A11) as

Ime−itln(x)Mitθ(τ)=Im∫u=0∞exp−uf1,r(t,θ(τ))exp−itln(x)+uf1,i(t,θ(τ))e−udu=−∫u=0∞exp−uf1,r(t,θ(τ))sintln(x)+uf1,i(t,θ(τ))e−udu.

By substituting this in (A11), we obtain the meta distribution ${\bar{F}}_{P_{r}\left(\tau \right)}\left(x\right)$ as

$$\begin{array}{cc} \; & =\frac{1}{2}-\frac{1}{\pi }\sum_{m=0}^{\infty }P\left({\tilde{M}}_{p}=m\right)\int_{t=0}^{\infty }\int_{u=0}^{\infty }\exp\left(-u\left(1+f_{1,r}\left(t,\theta \left(\tau \right)\right)\right)\right)\sin\left(t\ln\left(x\right)+uf_{1,i}\left(t,\theta \left(\tau \right)\right)\right)du\frac{dt}{t},\\ \; & \overset{\left(a\right)}{=}\frac{1}{2}-\frac{1}{\pi }\sum_{m=0}^{\infty }P\left({\tilde{M}}_{p}=m\right)\int_{t=0}^{\infty }\frac{f_{1,i}\left(t,\theta \left(\tau \right)\right)\cos\left(t\ln(x)\right)+\left(\left(f_{1,r}\left(t,\theta \left(\tau \right)+1\right)\sin\left(t\ln(x)\right)\right)\right)}{{\left(f_{1,r}\left(t,\theta \left(\tau \right)\right)+1\right)}^{2}+{\left(f_{1,i}\left(t,\theta \left(\tau \right)\right)\right)}^{2}}\frac{dt}{t}, \end{array}$$

where step (a) is obtained by apply $\sin\left(t\ln\left(x\right)+uf_{1,i}\left(t,\theta \left(\tau \right)\right)\right)=$ $\sin\left(t\ln\left(x\right)\right)\cos\left(uf_{1,i}\left(t,\theta \left(\tau \right)\right)\right)+\cos\left(t\ln\left(x\right)\right)\sin\left(uf_{1,i}\left(t,\theta \left(\tau \right)\right)\right)$ and then using the following integral identity

$$\int_{0}^{\infty }e^{-ax}\sin\left(bx\right)dx=\frac{b}{a^{2}+b^{2}},\;\int_{0}^{\infty }e^{-ax}\cos\left(bx\right)dx=\frac{a}{a^{2}+b^{2}}.$$

Solving further from step (a), we obtain the meta distribution of the rate coverage.
